# Grass Carp Follisatin: Molecular Cloning, Functional Characterization, Dopamine D1 Regulation at Pituitary Level, and Implication in Growth Hormone Regulation

**DOI:** 10.3389/fendo.2017.00211

**Published:** 2017-08-24

**Authors:** Roger S. K. Fung, Bai Jin, Mulan He, Karen W. Y. Yuen, Anderson O. L. Wong

**Affiliations:** ^1^School of Biological Sciences, University of Hong Kong, Hong Kong, Hong Kong

**Keywords:** follistatin, activin, dopamine, growth hormone, pituitary, grass carp

## Abstract

Activin is involved in pituitary hormone regulation and its pituitary actions can be nullified by local production of its binding protein follistatin. In our recent study with grass carp, local release of growth hormone (GH) was shown to induce activin expression at pituitary level, which in turn could exert an intrapituitary feedback to inhibit GH synthesis and secretion. To further examine the activin/follistatin system in the carp pituitary, grass carp follistatin was cloned and confirmed to be single-copy gene widely expressed at tissue level. At the pituitary level, follistatin signals could be located in carp somatotrophs, gonadotrophs, and lactotrophs. Functional expression also revealed that carp follistatin was effective in neutralizing activin’s action in stimulating target promoter with activin-responsive elements. In grass carp pituitary cells, follistatin co-treatment was found to revert activin inhibition on GH mRNA expression. Meanwhile, follistatin mRNA levels could be up-regulated by local production of activin but the opposite was true for dopaminergic activation with dopamine (DA) or its agonist apomorphine. Since GH stimulation by DA *via* pituitary D1 receptor is well-documented in fish models, the receptor specificity for follistatin regulation by DA was also investigated. Using a pharmacological approach, the inhibitory effect of DA on follistatin gene expression was confirmed to be mediated by pituitary D1 but not D2 receptor. Furthermore, activation of D1 receptor by the D1-specific agonist SKF77434 was also effective in blocking follistatin mRNA expression induced by activin and GH treatment both in carp pituitary cells as well as in carp somatotrophs enriched by density gradient centrifugation. These results, as a whole, suggest that activin can interact with dopaminergic input from the hypothalamus to regulate follistatin expression in carp pituitary, which may contribute to GH regulation by activin/follistatin system *via* autocrine/paracrine mechanisms.

## Introduction

Follistatin, a glycoprotein first isolated in porcine follicular fluid by its activity to inhibit follicle-stimulating hormone (FSH) secretion (1), can bind activin with high affinity and nullify its biological actions by preventing its binding and subsequent activation of type I and II activin receptors expressed at tissue level (2). The molecular structure of follistatin is highly conserved from fish to mammals (3). At tissue level, e.g., in the pituitary (4) and ovarian follicle (5), co-expression of follistatin and activin is frequently observed and follistatin expression is highly inducible by activin, which constitutes a local feedback for signal termination of activin actions (6). At cellular level, follistatin expression (e.g., in skeletal myoblast) is mediated *via* RSPO-LGR4 signaling (7) and Wnt/β-catenin pathway (8). In general, activin and its binding protein follistatin are widely expressed in various tissues and play a role in diverse functions including folliculogenesis (9), spermatogenesis (10), implantation and pregnancy (11, 12), regulation of pituitary hormones (13), energy metabolism (14), adipocyte differentiation (15), inflammation and immune responses (16), wound healing/tissue repair (17, 18), and stem cell survival during embryonic development (19). Aberrant expression/dysregulation of activin/follistatin system is also associated with carcinogenesis and tumorigenesis, e.g., in prostate cancer (20). Given that follistatin can also induce muscle growth and development *via* its binding with myostatin expressed in skeletal myoblasts (21), its potential application in “muscling”/growth promotion of farm animals has been a focus for recent development of agricultural biotechnology.

In mammals (e.g., rodents), the activin/follistatin system is a key component for reproductive function both in the pituitary (for gonadotropin regulation) and at the gonadal level (for oocyte/sperm maturation and steroid production) (5). In the rat pituitary, activin is expressed mainly in gonadotrophs (22) and stimulates FSH secretion and gene expression with parallel rise in GnRH receptor expression (23). The stimulatory effects of activin, however, could be inhibited by local production of follistatin (13), which is known to be originated mainly from folliculo-stellate cells (24), probably under the stimulatory influence of activin (25). In the same model, follistatin can also be located in gonadotrophs (26) and its expression could be up-regulated by GnRH and PACAP (4), implying that, besides the local action of activin, pituitary expression of follistatin is also under the influence of hypothalamic signals. Although activin in general is considered to have little/no effect on luteinizing hormone (LH), it is capable of inducing LHβ gene expression in pituitary cell lines (e.g., in LβT2 cells) (27) and parallel regulation of growth hormone (GH) and prolactin (PRL) release/gene expression has also been reported (e.g., in GH3 cells) (28, 29). At present, the functional role of follistatin in these “pituitary responses” is still unclear. In lower vertebrates, e.g., in fish models, the activin/follistatin system in the pituitary and its involvement in gonadotropin regulation appear to be well-conserved (30) and have been documented in the goldfish (31), zebrafish (32), and more recently in European eel (33). In fish species, interestingly, activin was found to have opposite effects on the two gonadotropins, with stimulation on FSH but inhibition on LH expression (31–33), and these differential effects could be blocked by follistatin at pituitary level, e.g., in zebrafish (32) and goldfish (34, 35). Of note, unlike the studies in mammals, cell-type-specific expression of follistatin has not been characterized in fish pituitary. Besides, hypothalamic regulation of pituitary follistatin as a means to modulate the functionality of activin/follistatin system has not been examined in fish models.

In grass carp, a representative member of carp family with high commercial value in Asian countries, our previous studies have shown that GH and LH released locally at pituitary level can play a role in autocrine/paracrine regulation of GH (36) and PRL synthesis and secretion (37). In grass carp pituitary, gonadotrophs exist in the form of cell clusters within the matrix of somatotrophs located in the proximal pars distalis (38) and local interactions between the two cell types are known to constitute an intrapituitary feedback for GH regulation (39). In this feedback loop, LH secretion from gonadotrophs can induce GH release and gene expression in neighboring somatotrophs *via* paracrine actions mediated by cAMP/PKA and JAK_2_/MAPK pathways (40). At somatotroph level, the stimulatory effects on GH expression can be further amplified by GH autoregulation *via* GH-induced GH release and GH gene expression mediated by JAK_2_/MAPK and JAK_2_/PI3K cascades (41) and GH released locally in turn can trigger signal termination *via* paracrine inhibition of LH secretion from nearby goanadotrophs (39). In our recent study, activin was also found to interact with GH and LH released locally at pituitary level to modulate GH secretion and gene expression in grass carp (42). In this case, the two forms of activin β subunits, activin βA and βB, were shown to be up-regulated by GH treatment in carp pituitary cells *via* JAK_2_/STAT_5_, MAPK, and PI3K/Akt pathways and local production of activin A and B in turn could suppress GH release, GH production, and GH gene expression with concurrent drop in GH gene transcription *via* SMAD_2_/SMAD_3_-dependent mechanisms (42). In the same study, both basal as well as GH-induced activin βA and βB gene expression were also suppressed by LH released locally in carp pituitary cells, suggesting that activin may constitute as a new facet of the intrapituitary feedback loop for GH regulation.

In fish pituitary, direct innervation by dopaminergic fibers originated from the hypothalamus is well-documented (43, 44) and dopaminergic regulation of GH and LH release at the pituitary level has been reported in goldfish (45), tilapia (46), and rainbow trout (43). In fish models, interestingly, dopamine (DA) is known to induce GH release *via* activation of DA D1 receptor but inhibit LH secretion *via* DA D2 receptor expressed at pituitary level, e.g., in goldfish (47). In our previous study with grass carp pituitary cells, GH secretion induced by DA D1 induction has also been demonstrated (38) but the role of DA in pituitary regulation of activin/follistatin system is still unclear. In this study, as a follow-up of our investigation on GH regulation by activin/follistatin system in carp model, grass carp follistatin was cloned and its expression at tissue level, especially in the pituitary, was characterized by RT-PCR, Northern blot, and LC/MS/MS. The functionality of carp follistatin in neutralizing the biological actions of activin was validated by protein expression followed by functional studies in mammalian cell lines. Using primary culture of grass carp pituitary cells, follistatin induction by activin, dopaminergic regulation of follistatin and activin, and follistatin modulation of GH expression under activin inhibition were examined at the pituitary level. Using a pharmacological approach, the receptor specificity for DA regulation of follisatin was elucidated and confirmed to be mediated through pituitary D1 receptor. To establish the link between follistatin and somatotroph function, the effects of DA D1 activation on follistatin expression induced by activin and GH treatment were also tested in carp somatotrophs enriched by density gradient centrifugation. Our studies for the first time provide evidence that the activin/follistatin system within the carp pituitary can be regulated by dopaminergic input *via* pituitary D1 receptor and contribute to GH regulation *via* autocrine/paracrine mechanisms.

## Materials and Methods

### Animals and Test Substances

Mixed sexes of grass carp (*Ctenopharyngodon idellus*) with body weight of 2.0–2.4 kg were obtained from local wholesale market and maintained in well-aerated aquaria at 20^o^C under 12-h light:12-h dark photoperiod for 10–14 days prior to experimentation. For tissue sampling and pituitary cell preparation, the fish were sacrificed by anesthesia in 0.05% MS222 (Sigma-Aldrich, St. Louis, MO, USA) followed by spinosectomy according to the protocol approved by the Committee for Animal Use in Teaching and Research at the University of Hong Kong (Hong Kong). For drug treatment, porcine GH, human chorionic gonadotropin (hCG), and recombinant proteins for human activin A, activin B, and follistatin were obtained from Sigma-Aldrich while DA, its non-selective agonist apomorphine (APO), and agonists/antagonists for DA D1/D2 receptor, including SKF77434, SKF83566, Ly171555, and sulpiride were acquired from Calbiochem (San Diego, CA, USA). The protein hormones, activin, and follistatin used in our studies were dissolved in double-distilled water as 1 mM stock solutions and stored frozen at −80^o^C in small aliquots. APO and D1/D2 drugs were prepared in a similar manner except that they were dissolved in DMSO as 10 mM stock solutions. During our experiments, except for DA which was freshly prepared right before drug treatment, frozen stocks of test substances were routinely diluted with prewarmed (28^o^C) culture medium to appropriate concentrations 10 min prior to drug administration. The final dilutions of DMSO were always maintained at levels ≤0.1% and did not affect target gene expression in our cell culture system.

### Molecular Cloning, Gene Copy Number, and Tissue Expression of Carp Follistatin

To establish the structural identity of grass carp follistatin, total RNA was isolated from the carp pituitary, reversely transcribed with SuperScript II (Invitrogen, Carlsbad, CA, USA) and used in 3′/5′RACE with primers designed based on the conserved regions of follistatin in goldfish and zebrafish. Full-length cDNA for carp follistatin was compiled using MacVector 9.5 (Oxford Molecular, Madison, WI, USA). Sequence alignment, protein modeling, and phylogenetic analysis of follistatin sequence obtained were conducted using CLUSTAL-W (http://www.ebi.ac.uk/Tools/msa/clustalw2), SWISS-MODEL (http://swissmodel.expasy.org/), and MEGA 6.0 (http://www.megasoftware.net), respectively. To study the gene copy number of follistatin in the carp genome, Southern blot was performed in genomic DNA isolated from the whole blood of grass carp (48) after digestion with restriction enzymes including Pvu II, Sty I, Bgl II, EcoR V, Xba I, BamH I, and Bgl I followed by hybridization with a DIG-labeled cDNA probe for follistatin.

For tissue expression profiling of follistatin, RT-PCR was carried out in total RNA prepared from selected tissues and brain areas with parallel PCR for β actin expression as the internal control. In this case, the RT samples prepared after DNAse I digestion and reverse transcription (RT) of total RNA obtained from target tissues/brain areas were subjected to PCR using primers for carp follistatin (5′TAAATGCTTAACCTCCAGGAGAA3′ and 5′ATCGCATGACTTGGCC TTGATG3′). PCR reactions were conducted for 35 cycles with denaturation at 94^o^C for 30 s, annealing at 58^o^C for 30 s, and extension at 72^o^C for 90 s, and the authenticity of PCR product (292 bp) was confirmed by PCR Southern using the DIG-labeled probe for follistatin used in the preceding section. To shed light on the size and form(s) of follistatin transcript(s) expressed at the pituitary level, total RNA prepared from the carp pituitary was size-fractionated in 1% agarose gel and used in Northern blot (37) with a DIG-labeled riboprobe for follistatin. To confirm the presence of an activin/follistatin system in the carp pituitary, protein expression of follistatin and activin was also examined using LC/MS/MS with pituitary lysate after trypsin digestion in a Sciex TripleTOF-5600 system (AB Sciex, Concord, ON, Canada) as described previously (49).

To test if follistatin also exhibits a cell-type-specific expression at the pituitary level similar to mammals, RT-PCR coupled with laser capture microdissection (LCM) of immuno-identified carp somatotrophs, lactotrophs, and gonadotrophs was also performed. In cytospin preparation of carp pituitary cells, the respective cell types were identified by immunostaining with antisera for carp GH (1:10,000), PRL (1:5,000), and LH (1:5,000) using a Vectastain ABC staining kit (Vector Laboratories, Burlingame, CA, USA) and isolated by LCM using a PixCell-II Cell Isolation System (Arcturus Engineering, Mountain View, CA, USA) (50). The target cells captured (~250 cells per sample) were subjected to DNase I digestion and RT followed by PCR using the same conditions with cycle number extended to 45 cycles with primers for follistatin as described in preceding section for tissue expression profiling. In this experiment, RT-PCR with RT sample prepared from mixed populations of pituitary cells was used as the positive control and PCR for β actin expression was used as the internal control.

### Protein Expression and Functional Testing of Carp Follistatin

To confirm that the newly cloned cDNA encodes target protein with bioactivity, expression of grass carp follistatin was performed in CHO cells. In this case, the open reading frame (ORF) of carp follistatin without 5′/3′ untranslated region (UTR) was PCR isolated and subcloned into the eukaryotic expression vector pcDNA3.1. Using lipofectamine (Invitrogen), the vector was transfected into CHO cells cultured in 35 mm dish with a seeding density of 0.5 × 10^6^ cells/dish. After a 6-h transfection in OPTI-MEM medium, the culture medium was replaced with DMEM medium supplemented with 10% FBS and the cell culture was maintained for 3 days under 5% CO_2_ and saturated humidity prior to the collection of conditioned medium containing follistatin released from CHO cells. To test for the functionality of follistatin expressed, αT3 cells with activin receptor expression (51) were maintained in DMEM medium in 24-well plates with a seeding density of 0.1 × 10^6^ cells/well and transfected for 6 h with lipofectamine in the presence of 0.2 μg/well of the firefly luciferase-expressing activin reporter pAR3-Lux (52), 0.05 μg/well FAST-2 expression vector (as a co-factor for activin induction), and 0.02 μg/well RL-TK.Luc (a renilla luciferase-expressing vector as internal control). After transfection, the cells were incubated for 18 h for recovery followed by drug treatment for 24 h with activin A (10 ng/ml) and B (10 ng/ml), respectively. To test the effect of follistatin in neutralizing activin’s actions, follistatin co-treatment by adding 200 μ1 volume of the conditioned medium with carp follistatin was performed in αT3 cells in the presence of activin A and B, respectively. After treatment, the cells were lysed with passive lysis buffer (Promega, Madison, WI, USA) and the lysate prepared was subjected to measurement of firefly and renilla luciferase activities using a Dual-Glo™ Luciferase Assay (Promega).

### Follistatin and Activin mRNA Expression in Grass Carp Pituitary Cells

Grass carp pituitary cells was prepared by trypsin/DNase digestion method (38) and seeded at a density of 2.5 × 10^6^ cell/well in poly-D-lysine precoated 24-well plates. After overnight incubation to allow for recovery from enzyme digestion, static incubation with activin A, activin B, follistatin, DA, and its non-selective agonist APO or its D1-/D2-specific agonists/antagonists was performed for the duration as indicated for individual experiments to study their effects on follistatin, activin βA, and activin βB mRNA expression at the pituitary level. To shed light on the role of follistatin on GH regulation by activin, parallel studies were also conducted with activin A and B induction with/without follistatin co-treatment to examine their effects on GH mRNA expression. To confirm that DA by acting through D1 receptor can play a role in follistatin expression at the somotroph level, carp somatotrophs (86–92% pure) were enriched from mixed populations of pituitary cells by Percoll density gradient centrifugation (53) and used in the testing of follistatin mRNA expression with D1 activation by the D1 agonist SKF77434 in the presence or absence of activin A, activin B, or GH co-treatment. In these experiments, total RNA was isolated with Trizol (Sigma-Alrich) after drug treatment, digested with DNAse I and reversely transcribed into RT samples for subsequent real-time PCR for follistatin, activin βA, activin βB, and/or GH mRNA measurement. For individual real-time PCR assays, quantitative PCR with primers for the respective gene targets was conducted in a RotorGene 6000 Real-time PCR System (Corbett, NSW, Australia) using a LightCycler^®^ 480 SYBR Green I Master Kit (Roche, Basel, Switzerland). For real-time PCR of follistatin, gene-specific primers for carp follistatin (5′ATGCAAAGGGCACCCGGATCT3′ and 5′ATCGCATGACTTGGCCTTGATG3′, producing a 292 bp PCR product with *Tm* value at 92^o^C) were used for quantitative PCR with serial dilutions of plasmid DNA carrying the ORF of follistatin as the standards for data calibration. PCR reactions were conducted with denaturation at 95^o^C for 30 s, annealing at 56^o^C for 30 s, and extension at 72^o^C for 30 s for a total of 32 cycles and PCR signals for follistation expression were recorded by the end of individual cycles with acquiring temperature at 88^o^C. For the measurement of activin βA, activin βB, and GH mRNA expression, real-time PCR for the respective gene targets was conducted as described previously (42). In our studies, the authenticity of PCR products was routinely monitored by melting curve analysis after the real-time PCR assays and parallel measurement of 18S RNA expression was used as the internal control.

### Data Normalization and Statistical Analysis

For expression studies of carp follistatin, the raw data for light emission caused by luciferase activity were registered in terms of relative light unit (in “RLU”). For normalization of potential variations in transfection efficiency between wells, the RLU data for firefly luciferase activity were normalized as a ratio of renilla luciferase activity detected in the same sample (referred to as “LUC activity ratio”). The normalized data were then expressed as a percentage of the mean value in the control group (as “%Ctrl”) and used for subsequent statistical analysis. For real-time PCR of target gene expression in carp pituitary cells, standard curves constructed with the respective plasmid DNA standards with a dynamic range of ≥10^5^, amplification efficiency ≥98%, and correlation coefficient ≥0.95 were used for data calibration under the unsupervised mode with RotorGene Q software 1.7 (Corbett). Since the levels of 18S RNA expressed in carp pituitary cells did not exhibit significant changes with drug treatment in our experiments, the raw data for target transcripts (in femtomole target detected per 10^6^ cells) were transformed as a percentage of the mean value in the control group without drug treatment (as “%Ctrl”). Data presented (mean ± SEM) are pooled results from four to six separate experiments (*N* = 4–6) and were analyzed with one-way (for dose dependence and interaction studies) or two-way ANOVA (for time course) followed by Newman–Keuls multiple comparison test. Differences between experimental groups were considered as significant at *P* < 0.05.

## Results

### Molecular Cloning and Structural Analysis of Grass Carp Follistatin

**Figure 1:**
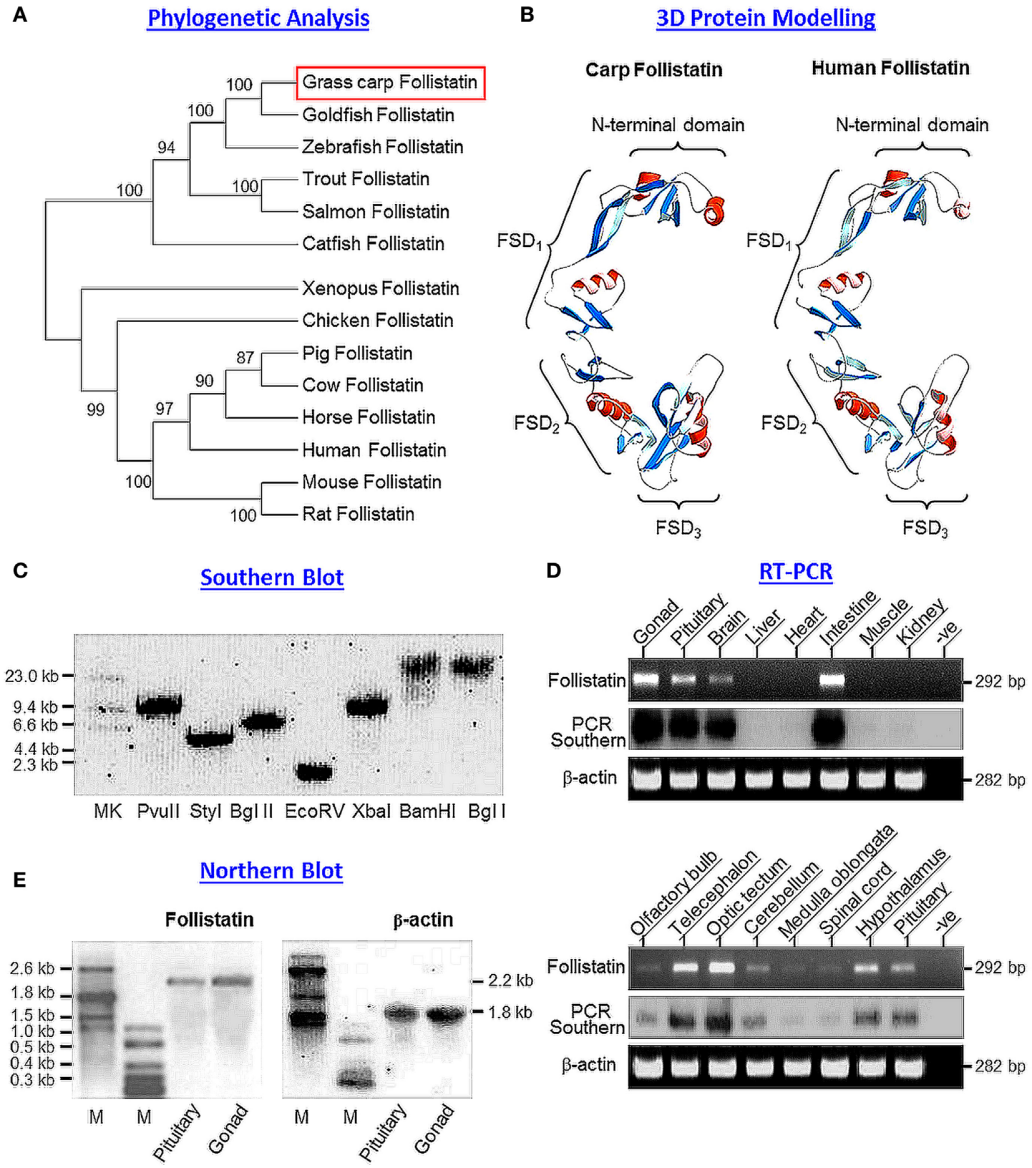
Sequence analysis, gene copy number, and tissue expression of grass carp follistatin. **(A)** Phylogenetic analysis of carp follistatin cDNA with corresponding sequences reported in other species using neighbor-joining method with MEGA 6.0. The numbers presented with the guide tree are the percentage of bootstrap values based on a 1,000 bootstraps. **(B)** 3D protein modeling of carp follistatin using SWISS-MODEL and DeepView with the crystal structure of human follistatin as the template. The regions in red and blue represent the helical motifs and β sheet structures, respectively, within the N-terminal and FSD_1–3_ domains. The four structural domains can be clustered into a “concave” structure, which is known to be essential for activin binding. **(C)** Gene copy number for carp follistatin deduced by genomic Southern. Southern blot was conducted with a DIG-labeled probe for follistatin in carp genomic DNA with prior digestion of restriction enzymes as indicated. **(D)** Tissue expression of follistatin in grass carp. Total RNA was isolated from selected tissues and brain areas as indicated and subjected to RT-PCR with primers specific for follistatin. The authenticity of PCR product was confirmed by PCR Southern with DIG-labeled probe for follistatin with parallel PCR for β actin as internal control. **(E)** Characterization of follistatin transcript in the carp pituitary. Total RNA was isolated from the carp pituitary, resolved in 1% agarose gel and subjected to Northern blot with DIG-labeled probe for follistatin. In this experiment, total RNA prepared form the gonad was used as a positive control and parallel blotting for β actin transcript was used as the internal control (M: size markers for RNA transcripts).

Using 5′/3′RACE, the full-length cDNA for grass carp follistatin (GenBank Accession No DQ340765) was cloned. The cDNA is 1,307 bp in size composed of a 72 bp 5′UTR, 266 bp 3′UTR, and 966 bp ORF encoding a 322 a.a. follistatin precursor with deduced MW of ~35.6 kDa (Figure S1 in Supplementary Material). Based on phylogenetic analysis using neighbor-joining method with MEGA 6.0, the newly cloned cDNA could be clustered into the clade of fish follistatin and closely related to follistatin sequences reported in other members of carp family including goldfish and zebrafish (Figure 1A). Within the 3′UTR, four polyadenylation signals (AAGAAA, ATTAAA, and two AATGAA) could be noted in close proximity/upstream region of the poly(A) tail. Based on structural analysis using SignalP 3.0 (http://cbs.dtu.dk/services/SignalP), a signal peptide with the size of 33 a.a. was identified in the N-terminal of the deduced protein of carp follistatin precursor. By sequence alignment with follistatin proteins reported in other species (Figure S2 in Supplementary Material), the four structural domains in the carp follistatin, namely the N-terminal domain followed by FSD_1_ (with a lysine-rich proteoglycan-binding sequence), FSD_2_, and FSD_3_ domains, as well as the 36 cysteine residues spreading within these four structural motifs were found to be highly conserved. Interestingly, the C-terminal domain reported in tetrapod species appeared to be missing in fish models. By comparing the protein sequences of follistatin, the sequence homology is >90% for fish species of the carp family (94–96% vs the sequences for goldfish and zebrafish) but the homology level is notably reduced when compared with the follistatin sequences of other fish models (71% vs the sequence in catfish) or tetrapods (70–74% for the sequences from amphibian to mammals). Despite the reduced level of sequence homology (only 71% vs the sequence in human), the 3D protein model deduced from carp follistatin, especially in the spatial orientation of helical motifs and β sheets in the N-terminal and FSD_1–3_ domains, was found to be highly comparable, if not identical to the corresponding structures in human follisatin (Figure 1B). Of note, the C-terminal domain forming the C-terminal tail of follistatin in tetrapods was not found in the corresponding region in carp model.

### Gene Copy Number and Tissue Expression of Carp Follistatin

To deduce the copy number of follistatin gene in carp genome, Southern blot was conducted in genomic DNA isolated from whole blood of grass carp. As shown in Figure 1C, a single band was detected in DNA samples predigested with Pvu II, Sty I, Bgl II, Ecor V, Xba I, BamH I, and Bgl I after hybridization with DIG-labeled probe for carp follistatin, confirming that the newly cloned follistatin is a single-copy gene in carp model. To shed light on tissue distribution of follistatin expression, RT-PCR was performed in selected tissues and brain areas in grass carp (Figure 1D). In this study, the PCR cycle number was fixed at 35 cycles, as it is in the mid-log phase of PCR amplification of follistatin. In our tissue expression profiling, follistatin PCR signals were detected at high levels in the gonad and intestine and with notable level in the brain and pituitary, but not in the liver, heart, muscle, and kidney. In the brain, follistatin was found to be ubiquitously expressed in different areas, with high levels in the telencephalon and optic tectum, to a lower extent in the hypothalamus, cerebellum, and pituitary, and at very low levels in the olfactory bulbs, medulla oblongata, and spinal cord. The lack of follistatin signals in the liver, heart, muscle, and kidney could not be due to RNA degradation during sample preparation, as β actin signal (as internal control) was consistently detected in all the samples examined.

Given that multiple transcripts of follistatin giving rise to two major isoforms of follistatin, follistatin-315 and follistatin-288, have been reported in mammals (54), the size and form(s) of follistatin mRNA expressed were also characterized in the carp pituitary using Northern blot with parallel study of the gonad as a positive control (Figure 1E). In this case, a single transcript for follistatin with 2.2 kb in size was detected both in the pituitary as well as in the gonad. To further examine protein expression of activin/follistatin system at pituitary level, LC/MS/MS was performed in trypsin-digested lysate prepared from the carp pituitary. Based on the mass spectra obtained, peptide fragments originated from carp follistatin (with protein coverage of 57.8%, Figure S3 in Supplementary Material), activin βA (with protein coverage of 56.9%, Figure S4 in Supplementary Material), and activin βB (with protein coverage of 59.4%, Figure S5 in Supplementary Material) could all be identified in this pituitary sample, indicating that the major components of activin/follistatin system are also expressed in the carp pituitary at protein level.

### Protein Expression and Functional Testing of Carp Follistatin

**Figure 2:**
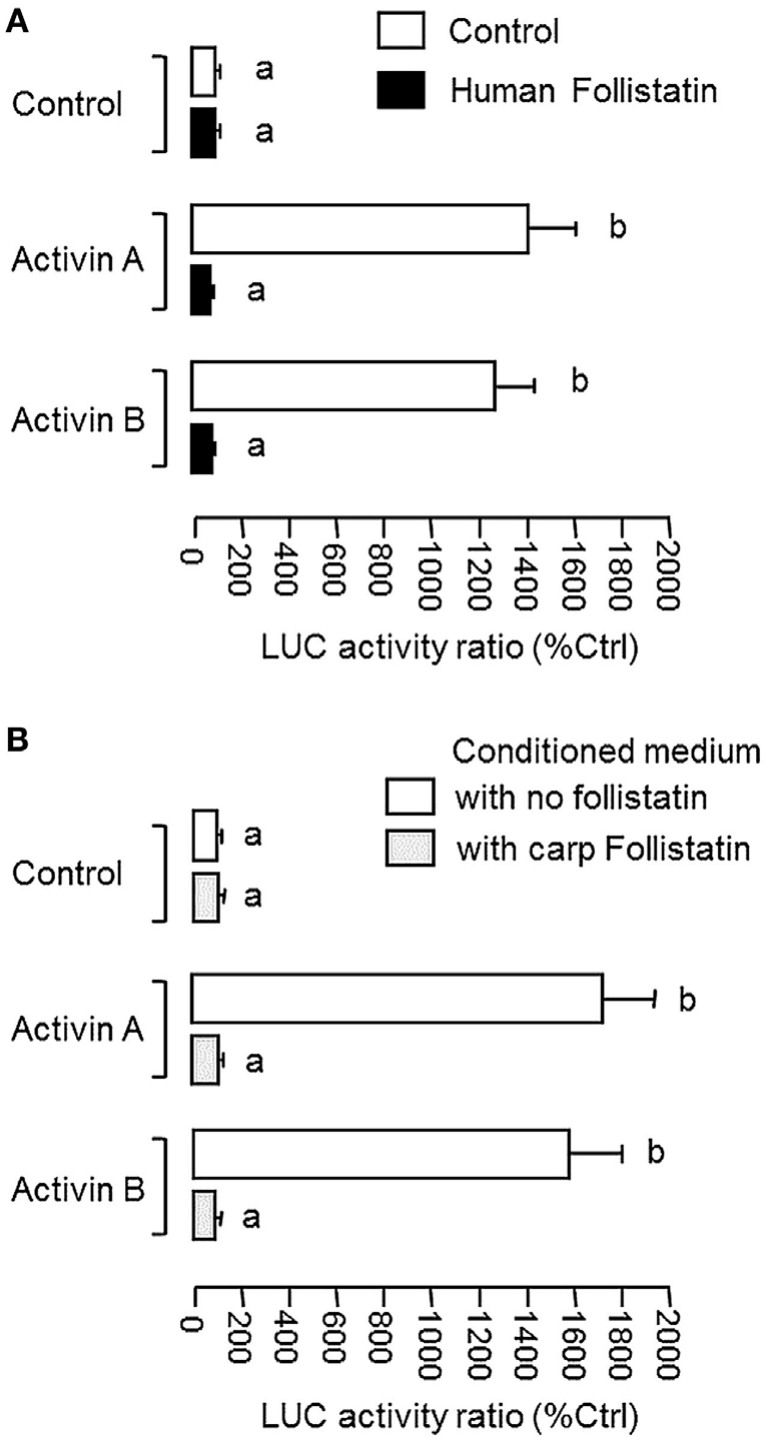
Functional characterization of carp follistatin in αT3 cells. **(A)** Validation of follistatin blocking of activin’s action in αT3 cells with pAR3-Lux transfection. In this experiment, αT3 cells were transfected with the pAR3-Lux reporter with 5′promoter carrying tandem repeats of activin-responsive elements and challenged for 24 h with human activin A (10 ng/ml) and B (10 ng/ml), respectively. In treatment group, activin A and B induction were conducted in the presence of human follistatin (100 ng/ml). **(B)** Grass carp follistatiin on activin-induced pAR3-Lux reporter activity expressed in αT3 cells. Conditioned medium obtained from CHO cells transfected with the expression vector for carp follistatin was used as the source of follistatin of carp origin. For functional testing of carp follistatin, αT3 cells transfected with pAR3-Lux were challenged for 24 h with activin A (10 ng/ml) and B (10 ng/ml) in the presence of the conditioned medium containing carp follistatin. Parallel incubation with conditioned medium harvested from CHO cells transfected with the blank vector pcDNA3.1 was used as the control treatment. In these experiments, cell lysate was prepared from αT3 cells after activin treatment (with/without follistatin) and used for luciferase activity measurement with a Dual-Glo^®^ assay kit. Data presented are expressed as mean ± SEM (*N* = 6) and the groups denoted by different letters represent a significant difference at *P* < 0.05 (ANOVA followed by Newman–Keuls test).

To confirm that the newly cloned cDNA indeed encodes follistatin with the ability to block activn’s actions, functional expression was conducted in CHO cells after transfection with the expression vector for carp follistatin. After 3-day culture to allow for the accumulation of carp follistatin released from CHO cells, the conditioned medium was harvested and their bioactivity was tested in αT3 cells transfected with the luciferase-expressing activin reporter pAR3-Lux (52) carrying tandem repeats of activin-responsive elements in its 5′ promoter. The αT3 cells were used as it is a pituitary cell line with activin receptor expression and known to be highly responsive to activin stimulation (51). In our study, treatment with human activin A (10 ng/ml) and B (10 ng/ml) were both effective in elevating luciferase activity expressed in αT3 cells and these stimulatory effects could be abolished by co-treatment with human follistatin (100 ng/ml, Figure 2A), implying that our assay system is fully functional in probing follistatin inhibition on activin actions. In parallel experiment with αT3 cells transfected with pAR3-Lux, incubation with conditioned medium containing carp follistatin released from CHO cells could also trigger a total blockade on the stimulatory effects of activn A (10 ng/m) and B (10 ng/ml) on luciferase activity expression (Figure 2B). However, similar treatment with conditioned medium harvested from CHO cells transfected with the blank vector (used as negative control) was found to have no effects on luciferase activity induced by activin A and B, respectively.

### Regulation of Activin/Follistatin System in Carp Pituitary Cells

**Figure 3:**
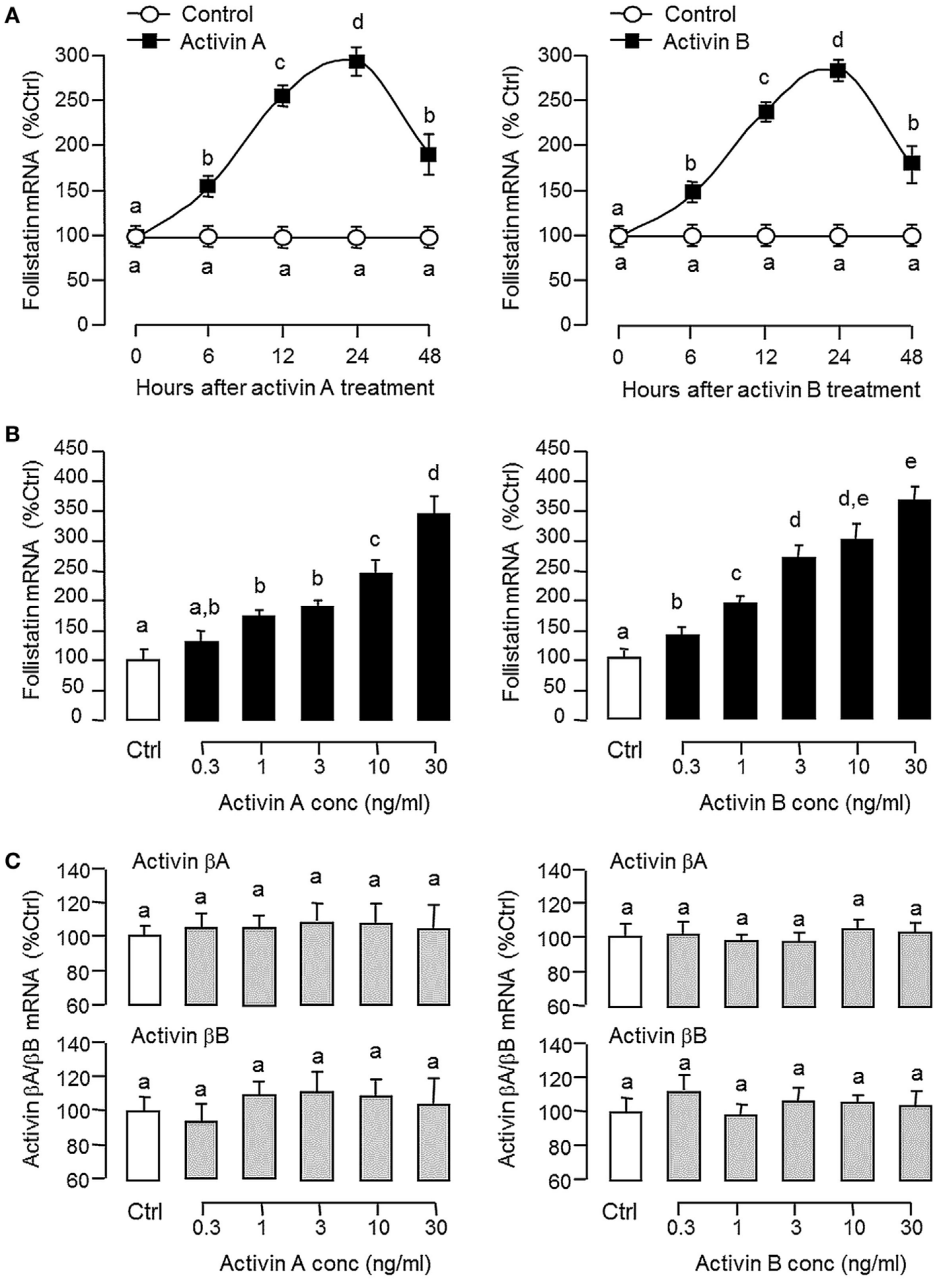
Effects of activin A and B on follistatin, activin βA, and activin βB expression in grass carp pituitary cells. **(A)** Time course and **(B)** dose dependence of activin-induced follistatin mRNA expression. **(C)** Dose dependence of activin treatment on activin βA and βB mRNA expression. In time course experiment, pituitary cells were incubated with activin A (10 ng/ml) or activin B (10 ng/ml) for the duration as indicated up to 48 h. To test for dose dependence, the duration of drug treatment was fixed at 24 h with pituitary cells challenged with increasing levels of activin A or B, respectively. After activin treatment, total RNA was isolated and used for real-time PCR of follistatin, activin βA, and activin βB mRNA, respectively. Data presented are pooled from four separate experiments (*N* = 4) and the groups denoted by different letters represent a significant different at *P* < 0.05 (ANOVA followed by Newman–Keuls test).

**Figure 4:**
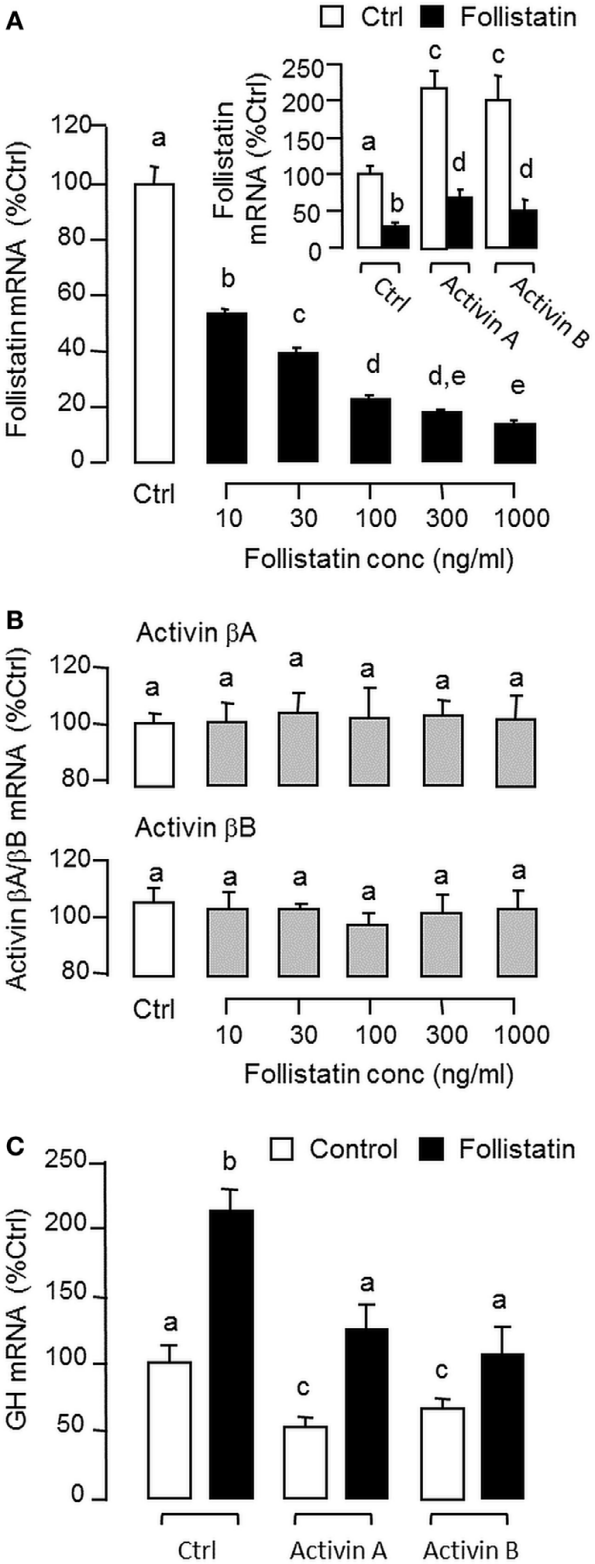
Removal of endogenous activin by follistatin on follistatin, activin βA, and activin βB expression in grass carp pituitary cells. **(A)** Effects of follistatin treatment on basal and activin-induced follistatin mRNA expression. **(B)** Effects of follistatin treatment on activin βA and βB mRNA expression. **(C)** Effects of follistatin treatment on basal and activin-induced growth hormone (GH) mRNA expression. To examine the effect on basal expression of follistatin mRNA, pituitary cells were treated for 24 h with increasing doses of follistatin as indicated. To test the effects of follistatin on activin modulation of follistatin and GH gene expression, pituitary cells were challenged for 24 h with activin A (10 ng/ml) or B (10 ng/ml) with/without the co-treatment of follistatin (100 ng/ml). In these experiments, total RNA was isolated after drug treatment and subjected to real-time PCR of follistatin, activin βA, activin βB, and GH mRNA, respectively. Data presented are pooled from four separate experiments (*N* = 4) and the groups denoted by different letters represent a significant different at *P* < 0.05.

To shed light on autoregulation of activin/follistatin system in the carp pituitary, the effects of activin treatment on follistatin and activin gene expression were tested in primary culture of grass carp pituitary cells. In this case, static incubation with activin A (10 ng/ml) and B (10 ng/ml) were both effective in inducing follistatin mRNA expression in a time-dependent manner with peak responses at 24 h (Figure 3A). By fixing the duration of drug treatment at 24 h, increasing doses of activin A (0.3–30 ng/ml) and B (0.3–30 ng/ml) could also elevate follistatin mRNA levels in a concentration-related fashion (Figure 3B) but with no corresponding changes in activin βA and βB transcript expression (Figure 3C). Given that follistatin produced locally is known to bind and neutralize activin’s actions at the pituitary level (13), removal of endogenous activin by static incubation with follistatin (10–1,000 ng/ml) was also performed in carp pituitary cells. As shown in Figures 4A,B, follistatin treatment could dose-dependently suppress follistatin mRNA levels but with no effects on activin βA and βB gene expression. In parallel experiment, co-treatment with follistatin (100 ng/ml) was also effective in blocking follistatin mRNA expression induced by activin A (10 ng/ml) and B (10 ng/ml), respectively (Figure 4A, inset). In the same study, GH mRNA level could be up-regulated by follistatin but the inhibitory effects on GH gene expression induced by activin A and B were also reverted/negated by follistatin co-treatment in carp pituitary cells (Figure 4C).

**Figure 5:**
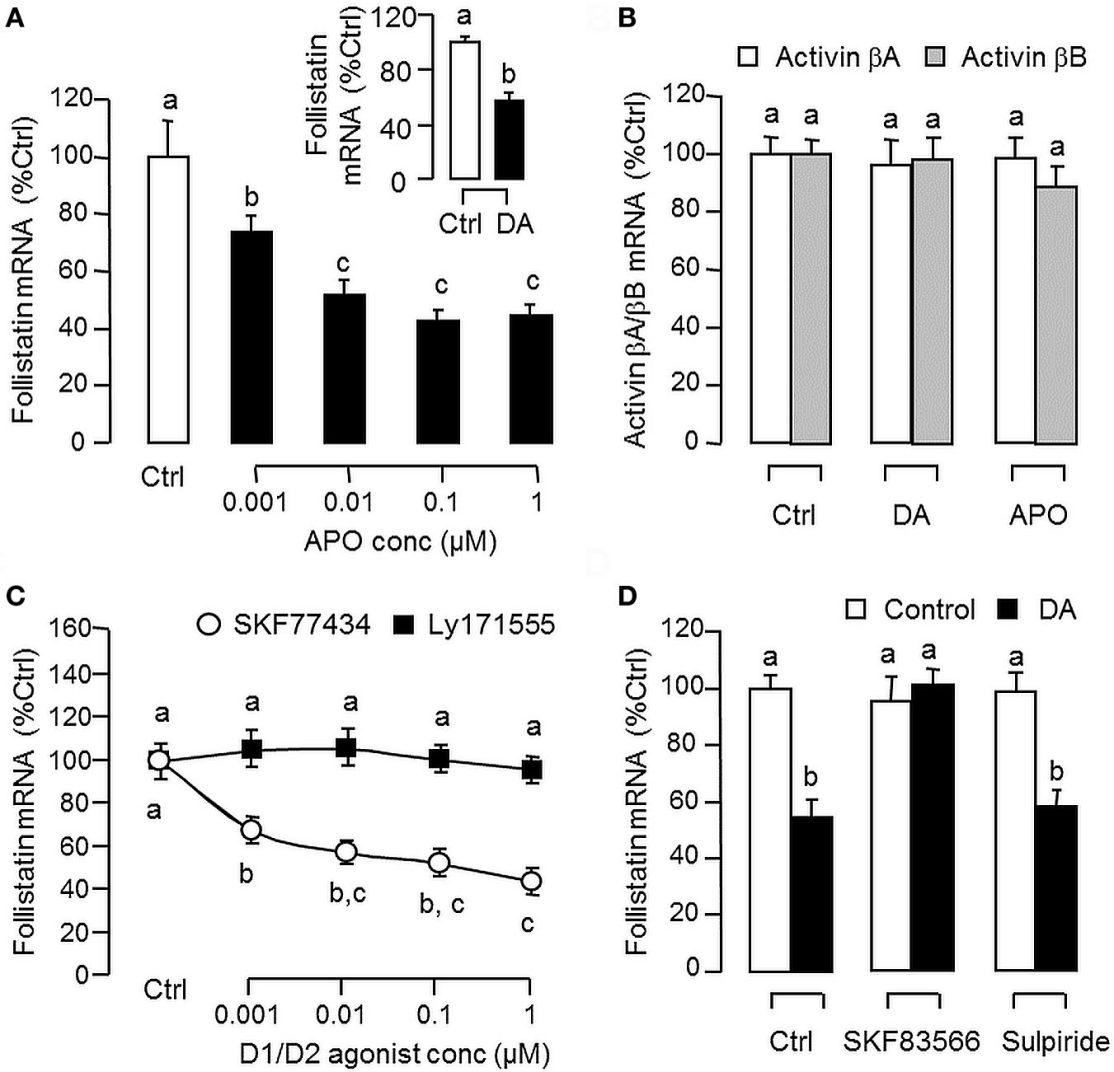
Dopaminergic regulation of follistatin and activin expression in carp pituitary cells. **(A)** Effects of dopamine (DA) and its non-selective agonist apomorphine (APO) on follistatin mRNA expression. Pituitary cells were treated for 24 h with increasing doses of APO (0.001–1 μM) or with a single dose of DA (1 μM). **(B)** Effects of DA and APO on activin βA and βB mRNA expression. Pituitary cells were treated for 24 h with DA (1 μM) or APO (1 μM). **(C)** Effects of DA D1 and D2 agonists on follistatin mRNA expression. Pituitary cells were treated for 24 h with increasing doses of the DA D1 agonist SKF77434 or D2 agonist Ly171555 as indicated. **(D)** DA D1 and D2 antagonists on the inhibitory effect of DA on follistatin mRNA expression. Pituitary cells were challenged with DA (1 μM) for 24 h in the presence or absence of the DA D1 antagonist SKF83566 (5 μM) or D2 antagonist sulpiride (5 μM). In these studies, total RNA was isolated after drug treatment and subjected to real-time PCR measurement for follistatin, activin βA, and activin βB mRNA, respectively. Data presented are pooled from four experiments (*N* = 4) and the groups denoted by different letters represent a significant different at *P* < 0.05.

**Figure 6:**
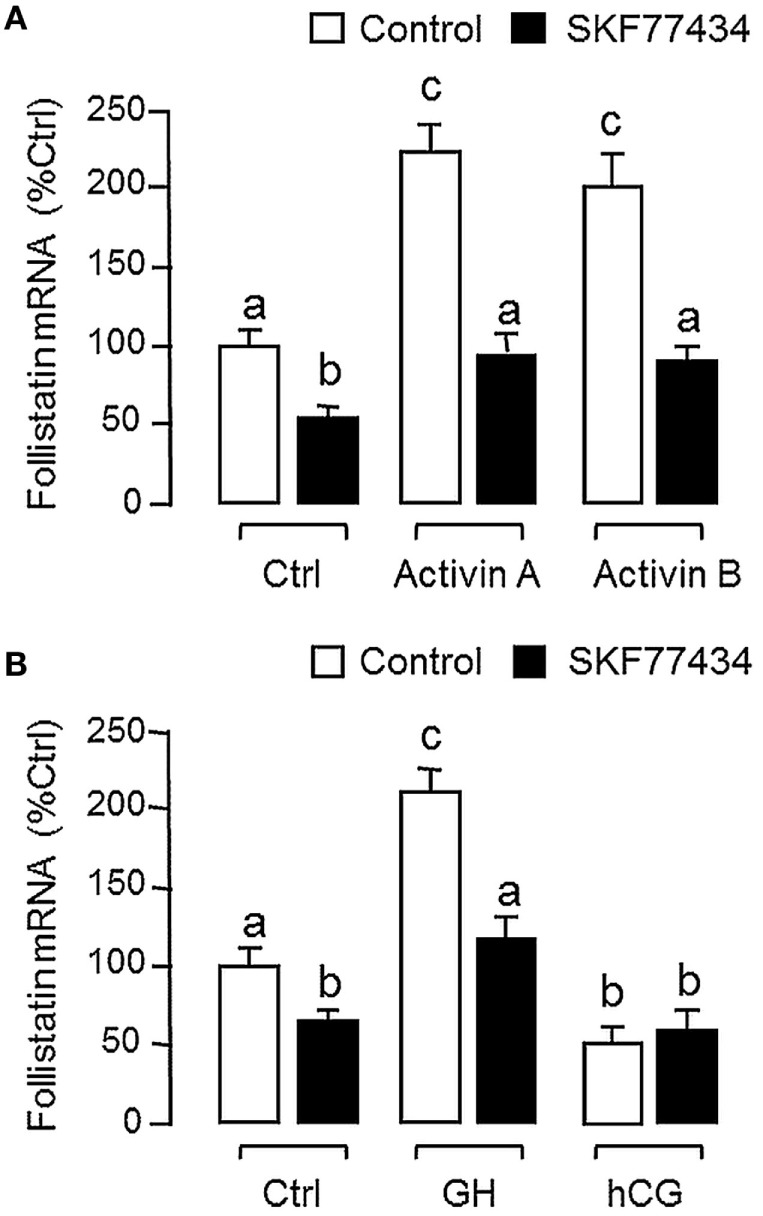
Follistatin regulation by activin, growth hormone (GH), and human chorionic gonadotropin (hCG) and their interaction with dopaminergic D1 stimulation in grass carp pituitary cells. **(A)** Dopamine (DA) D1 activation on activin-induced follistatin mRNA expression. Pituitary cells were incubated for 24 h with activin A (10 ng/ml) or B (10 ng/ml) in the presence or absence of the D1 agonist SKF77434 (1 μM). **(B)** DA D1 activation on the differential effects of GH and hCG on follistatin mRNA expression. Pituitary cells were exposed to GH (30 ng/ml) or hCG (30 IU/ml) for 24 h with/without the co-treatment of the D1 agonist SKF77434 (1 μM). After drug treatment, total RNA was isolated and subjected to real-time PCR for follistatin mRNA measurement. Data presented are pooled from four experiments (*N* = 4) and the groups denoted by different letters represent a significant different at *P* < 0.05.

Since dopaminergic regulation of pituitary functions is well-documented (55), it raises the possibility that the activin/follistatin system in the carp pituitary may also serve as a regulatory target for dopaminergic input form the hypothalamus. To test the idea, carp pituitary cells were challenged for 24 h with DA (1 μM) or its non-selective agonist APO (1–1,000 nM). In both cases, dopaminergic activation was effective in inhibiting follistatin mRNA levels (Figure 5A) without affecting activin βA and βB gene expression (Figure 5B). To further investigate the receptor specificity for DA action, carp pituitary cells were treated for 24 h with increasing levels (1–1,000 nM) of the DA D1 agonist SKF77434 and D2 agonist Ly171555, respectively (Figure 5C). In this study, dopaminergic inhibition on follistatin mRNA expression was mimicked in a dose-dependent manner by the D1 agonist SK77434 but not the D2 agonist Ly171555. In agreement with these results, the drop in follistatin mRNA level induced by DA (1 μM) could also be blocked by co-treatment with the D1 antagonist SKF83566 (5 μM) but not D2 antagonist sulpiride (5 μM) (Figure 5D). To examine DA D1 interaction with activin on follistatin regulation at pituitary level, carp pituitary cells were challenged for 24 h with activin A (10 ng/ml) and B (10 ng/ml) in the presence of the D1 agonist SKF77434 (1 μM). As shown in Figure 6A, follistatin mRNA expression induced by activin A and B could be notably suppressed by SKF77434 co-treatment. In our recent study, follistatin gene expression in the carp pituitary was found to be differentially regulated by GH and LH, with stimulation by GH but inhibition by LH (42). Therefore, DA D1 interactions with the two hormones were also investigated (Figure 6B). In carp pituitary cells, follistatin mRNA level could be up-regulated by GH induction (30 ng/ml) but this stimulatory effect was significantly reduced by co-treatment with SKF77434 (1 μM). The corresponding inhibition caused by hCG (30 IU/ml, used as a functional analog of LH), however, was found to be not additive to that of SKF77434.

### Follistatin Expression and Regulation in Carp Somatotrophs

**Figure 7:**
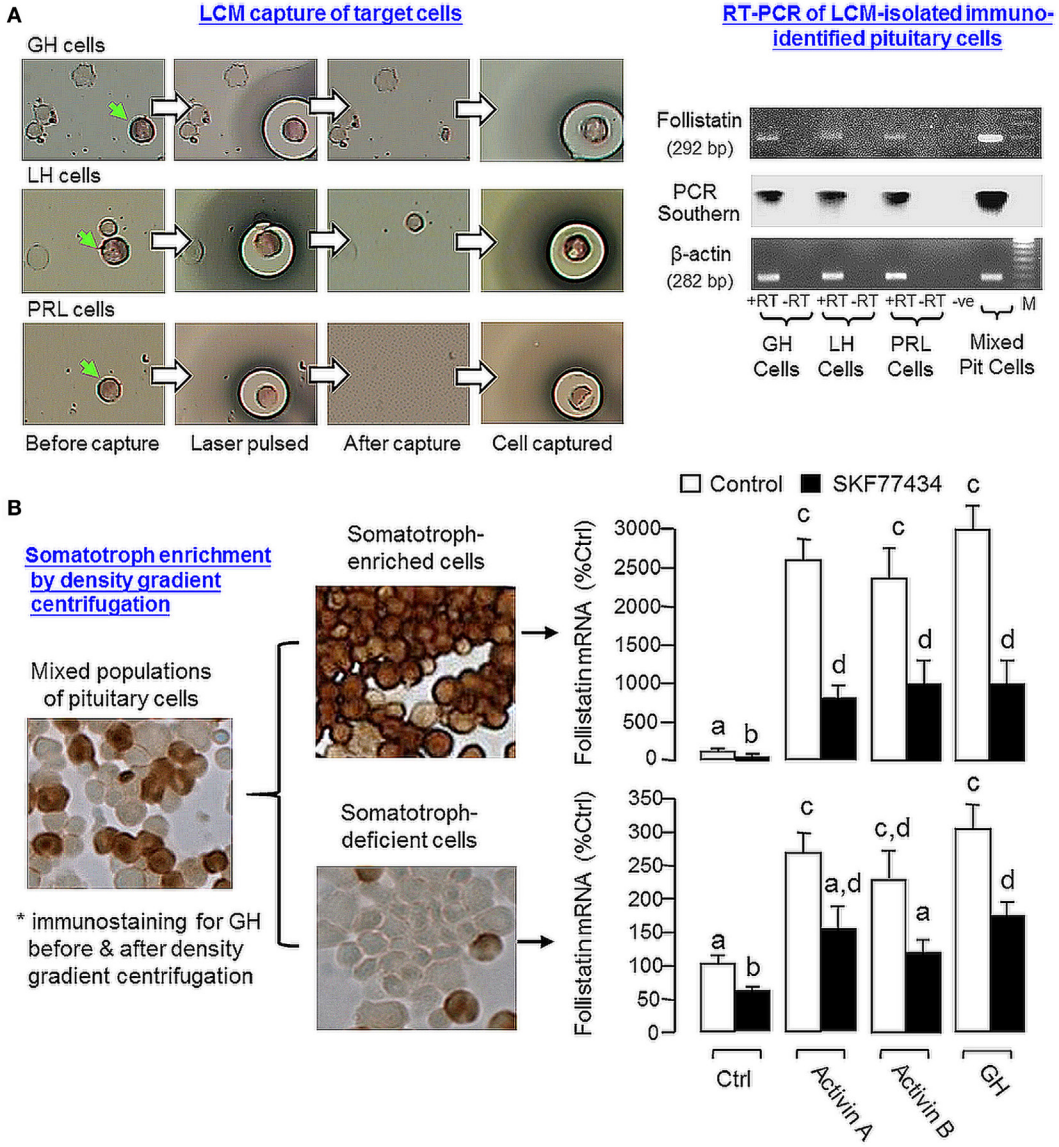
Effects of dopamine (DA) D1 activation on follistatin expression induced by activin and growth hormone (GH) in carp somatotrophs. **(A)** Follistatin expression in carp somatotrophs (GH cells), lactotrophs [prolactin (PRL) cells], and gonadotrophs [luteinizing hormone (LH) cells]. The three cell types (marked by green arrows) were identified by immunostaining using antisera for carp GH, PRL, and LH, respectively, and isolated on HS caps using laser capture microdissection technique. After that, RT-PCR was performed on the cells captured (~200 cells for individual cell types) using primers for follistatin. Mixed populations of pituitary cells were used as a positive control and RNA samples prepared with/without reverse transcription (±RT) were used to control for potential contamination with genomic DNA. The authenticity of PCR product was confirmed by PCR Southern using DIG-labeled probe for carp follistatin and parallel PCR for β actin mRNA was used as the internal control. **(B)** DA D1 activation on activin- and GH-induced follistatin mRNA expression in enriched somatotrophs. Enriched somatotrophs (86–92% pure) were prepared from mixed populations of carp pituitary cells by Percoll gradient centrifugation and the leftover cells (with only 3–4% somatotrophs, referred to as the “somatotroph-deficient” cells) were also harvested to serve as a parallel control. The two cell fractions were treated with activin A (10 ng/ml), activin B (10 ng/ml), and GH (30 ng/ml) for 24 h in the presence or absence of the D1 agonist SKF77434 (1 μM). After that, total RNA was isolated and used for real-time PCR for follistatin mRNA measurement. Data presented are pooled from four experiments (*N* = 4) and the groups denoted by different letters represent a significant different at *P* < 0.05.

Given that follistatin is known to express in the pituitary of mammals in a cell-type-specific manner, e.g., in rodents (13), the expression profile of follistatin in various cell types in the carp pituitary was also characterized using RT-PCR coupled to LCM isolation of carp somatotrophs (GH cells), gonadotrophs (LH cells), and lactotrophs (PRL cells) identified by immunostaining with the respective antisera (Figure 7A). Similar to the result based on mixed populations of pituitary cells (“Mixed Pit cells”), the PCR signal for follistatin could also be detected in carp somatotrophs, gonadotrophs, and lactotrophs. These PCR signals could not be the result of genomic DNA contamination, as parallel PCR with RNA samples without (“-RT”, used as negative control) did not produce any PCR product in our study. To further examine follistatin regulation at somatotroph level, enriched fraction of carp somatotrophs (86–92% pure) was prepared from mixed populations of carp pituitary cells using Percoll density gradient centrifugation (53). The remaining cells after somatotroph enrichment (designated as “somatotroph-deficient cells”) were used as a parallel control for our experiments with carp somatotrophs (Figure 7B). In the two cell fractions prepared, follistatin mRNA levels could be up-regulated by 24-h treatment with activin A (10 ng/ml), activin B (10 ng/ml), and GH (30 ng/ml), respectively, with much higher responses in enriched somatotrophs (23–28-fold increase vs basal) compared with somatotroph-deficient cell fraction (only 2.2–3.0-fold increase vs basal). Similar to the results based on mixed populations of pituitary cells (Figure 6), the stimulatory effects on follistatin gene expression caused by activin and GH detected in the two cell fractions were notably suppressed by the D1 agonist SKF77434 (1 μM).

## Discussion

Activin/follistatin system is known to play a role in pituitary functions, e.g., for FSH (13), GH (28), and PRL secretion/gene expression (29) and its involvement in FSH regulation is also well-conserved in fish species, e.g., goldfish (31), zebrafish (32), and European eel (33). Unlike the extensive studies in mammals, not much is known for the pituitary regulation of activin/follistatin system in fish models. Recently, using grass carp as a representative for Cyprinid species, we have shown that local production of GH could induce activin βA and βB expression in carp pituitary cells and subsequent production of activin, interestingly, was found to inhibit GH secretion and GH gene expression (42), implying that activin can exert a negative feedback on GH regulation in carp pituitary. To further characterize the role of activin/follistatin system in GH regulation in carp species, grass carp follistatin was cloned and confirmed to be a single-copy gene in carp genome. Based on phylogenetic analysis and sequence alignment, the newly cloned cDNA was shown to be an ortholog of follistatin in fish species and closely related to the corresponding sequences in goldfish and zebrafish. As shown by 3D protein modeling, the four structural domains of carp follistatin, namely the N-terminal domain and FSD_1–3_ domains, can be clustered into a concave structure, which is known to be essential for activin binding (56), and the spacing and orientation of helical motifs and β sheets within these structural domains are also highly comparable if not identical to the human counterparts, implying that the 3D protein structure of follistatin, especially the “core structure” for activin binding, is highly conserved from fish to mammals. Of note, the C-terminal tail reported in the follistatin of tetrapods was found to be missing in carp follistatin. In mammals, two forms of follistatin, follistatin-315 (with C-terminal tail and mainly found in blood) and follistatin-288 (without C-terminal tail and mainly found at tissue level), have been identified and known to be formed by alternative splicing of exon 6 encoding the C-terminal domain (54). The C-terminal tail, which is Glu-rich and highly acidic, can interact with the proteoglycan-binding motif within FSD_1_ domain and reduce follistatin binding to the heparin chain of cell surface proteoglycan, and presumably, can allow for the release of follistain-315 into systemic circulation (57). At the pituitary level (e.g., in rat), follistatin-288 was found to be more potent than follistatin-315 in inhibiting FSH secretion (58), implying that the C-terminal tail can also affect follistatin function mediated by activin binding. In our study, the lack of C-terminal tail in carp follistation suggests that it is the functional equivalence of follistatin-288 and may exert its effects mainly at tissue level but not as an endocrine factor.

Regarding the autocrine/paracrine actions of follistatin, it is well-documented that follistatin produced locally can bind activin with high affinity and nullify/neutralize its biological effects by preventing its binding to activin receptors (2). In our study with functional testing in αT3 cells, grass carp follistatin expressed in CHO cells was shown to block the stimulation of activin A and B on target promoter with tandem repeats of activin-responsive elements originated from the human Mix2 promoter (52), implying that the newly cloned cDNA indeed encodes functional protein which can serve as a negative regulator for activin’s actions. In mammals, follistatin is expressed at high level in the gonad and plays a role in folliculogenesis (9), spermatogenesis (10), and sex steroid production (59). Besides the gonad, extragonadal expression of follistatin has also been reported, e.g., in the brain, pituitary, kidney, and heart (60, 61). In grass carp, high levels of follistatin expression could be located in the gonad and intestine and to a lower extent in various brain areas and pituitary. In general, the pattern of follistatin expression in grass carp is comparable with that reported in other fish species, e.g., in goldfish (34). Despite a lack of information for testicular function of follistatin in fish models, high level of gonadal expression of follistatin in carp species is in agreement with the role of activin/follistatin system in folliculogenesis and oocyte maturation reported in fish ovary, e.g., in zebrafish (30, 62). The functional role of follistatin in the intestine is still unclear, although activin is known to be involved in wound healing and inflammation in gut epithelium (63) and follistatin expression in rat intestinal epithelial cells can be inhibited by PPARγ in mammalian models (64). Within the brain of grass carp, follistatin was found to be widely expressed and with high levels of signals detected in the telencephalon and optic tectum, to a lower extent in the hypothalamus and cerebellum, and at low levels in olfactory bulbs, medulla oblongata, and spinal cord. The biological relevance of follistatin in these brain areas has yet to be examined in fish model, but in rodents, activn and follistatin are known to be expressed in the brain and play a role in neuronal development in the hippocampus (65) and adult neurogenesis after brain injury (66). In chick embryo, follistatin is also involved in segmental patterning of the hindbrain development (67) suggesting that the activin/follistatin system may be an integral component for the process of neuron development and organization within the central nervous system.

Although the pituitary actions of activin/follistatin system is well-documented in mammals (see Introduction for details), the corresponding effects in the fish pituitary appear to be similar and yet distinct from that of the mammals. For examples, activin is known to exert a stimulatory action on FSH but with little/no effect on LH secretion/gene expression, especially in primary culture of rat pituitary cells (23). In pituitary cells from fish species, e.g., in goldfish (31), zebrafish (32), and more recently in European eel (33), activin treatment can exert differential effects on the two gonadotropins, with stimulation on FSH but inhibition on LH expression. Similar to mammals (e.g., in rat pituitary cells) (26), follistatin expression in fish pituitary cells can be up-regulated by activin (34), and the differential effects of activin on the two gonadotropins can be blocked by follistatin, e.g., in goldfish (35), suggesting that a local feedback of activin/follistatin system is present in the fish pituitary and contributes to gonadotropin regulation. In grass carp, together with activin βA and βB mRNA detected in our cell culture experiments, follistatin was found to be expressed at the pituitary level with a single transcript of 2.2 kb in size. Using LC/MS/MS, protein expression of follistatin, activin βA, and activin βB was also confirmed in carp pituitary lysate, indicative of the presence of an activin/follistatin system in the carp pituitary. In the pituitary of mammals (e.g., in rat), activin is expressed mainly in gonadotrophs (22) while follistatin can be located in folliculo-stellate cells and to a lower extent in gonadotrophs (24). In our recent study using RT-PCR coupled with LCM isolation of immuno-identified pituitary cells, activin βB, the dominant form of activin expressed in the carp pituitary, was shown to be highly expressed in gonadotrophs and with a lower level in lactotrophs but not in somatotrophs, whereas activin βA was expressed in pituitary cells other than the three cell types examined (42). In this study, using a similar approach, follistatin expression could be identified in carp gonadotrophs, lactotrophs, and somatotrophs, implying that individual components of activin/follistatin system are expressed in a cell-type-specific manner within the carp pituitary. In carp pituitary cells, follistatin mRNA expression could be up-regulated by activin A and B, while the opposite was true by removing endogenous activin with follistatin. Besides, follistatin treatment also blocked follistatin gene expression induced by activin A and B. Of note, activin and follistatin were not effective in altering activin βA and βB mRNA levels at pituitary level, implying that follistatin may serve as a major regulatory target for the activin/follistatin system in carp species. In the carp pituitary, our recent study has revealed that (i) activin βA and βB expression could be induced by GH and (ii) activin A and B could suppress GH release and GH gene expression (42). In the current study, follistatin alone could elevate GH mRNA level in carp pituitary cells but the inhibitory effects of activin A and B on GH gene expression could be negated by follistatin co-treatment. These findings, as a whole, indicate that a local feedback *via* activin induction of follistatin also exists in carp pituitary and plays a role in GH regulation by activin/follistatin system in carp species.

In mammals, follistatin expression at pituitary level is known to be under the influence of hypothalamic factors. For examples, GnRH and PACAP can down-regulate FSHβ mRNA level by stimulating follistatin expression in rat gonadotrophs and LβT2 cell line (68). However, similar study on hypothalamic regulation of pituitary activin/follistatin system is still lacking in fish models. In bony fish, the anterior pituitary is under the direct innervations of a preoptico-hypophyseal dopaminergic pathway, e.g., in goldfish (44) and rainbow trout (43). Our previous studies in goldfish also reveal that DA can induce GH secretion *via* activation of pituitary D1 receptor (45) with concurrent inhibition on LH release *via* D2 receptor activation (47). Given that DA D1 induction of GH release has also been demonstrated in carp pituitary cells (38) and local production of activin can modulate GH release and GH gene expression in carp pituitary (42), we speculate that dopaminergic input at the pituitary level may have an effect on activin/follistatin system related to GH regulation in carp model. This idea is supported by our findings that follistatin mRNA levels in carp pituitary cells could be down-regulated by DA. Apparently, the inhibitory effects observed were mediated by DA D1 receptor activation in the carp pituitary, as (i) down-regulation of follistatin mRNA levels could be mimicked by D1 but not D2 agonist and (ii) DA inhibition on follistatin gene expression was blocked by D1 antagonist but D2 antagonist was not effective in this regard. Since dopaminergic stimulation was not effective in altering activin βA and βB mRNA expression in carp pituitary cells, the possibility of D1 regulation of follistatin expression *via* modifications of activin production is unlikely. Of note, follistatin mRNA expression induced by activin A and B could be blocked by D1 agonist, suggesting that the local feedback of activn/follistatin system *via* activin-induced follistatin expression is also under the negative regulation by DA D1 action. At the pituitary level (e.g., rat), DA D2 activation has been reported to inhibit lactotroph proliferation *via* local production of TGFβ, a family member of activin (69). To our knowledge, DA D1 regulation of activin/follistatin system has not been documented in mammalian models.

In our recent study, GH and LH released locally were shown to have differential actions on follistatin expression in carp pituitary cells, with stimulation by GH but inhibition by LH, and the stimulatory effect of GH was mediated by its paracrine induction on activin production (42), which constitutes a local regulation of actvin/follistatin system by pituitary hormones in grass carp. In our study with the same animal model, follistatin mRNA level was up-regulated by GH at pituitary cell level and the opposite effect was noted with hCG treatment. Furthermore, GH-induced follistatin gene expression was negated by D1 activation but the corresponding inhibition by hCG was not additive to that caused by D1 agonist. At present, the role of DA D1 action in LH regulation of actvin/follistatin system is still unclear. However, judging from DA D1 induction of GH release in our previous report (38) and our current findings of D1 inhibition of follistatin expression induced by GH and follistatin blockade of activin-inhibited GH gene expression, it would be logical to conclude that dopaminergic input in carp pituitary *via* D1 receptor activation can modulate the expression as well as pituitary actions of GH *via* activin/follistatin system. In our study with LCM isolation of pituitary cells identified by immunostaining, follistatin expression could be located in carp somatotrophs. In enriched faction of carp somatotrophs, the magnitude of follistatin mRNA expression induced by activin A, activin B, and GH (>20-fold vs control) was found to be much higher than the corresponding responses in “somatotroph-deficient” cell fraction or mixed populations of carp pituitary cells (~2–3-fold vs control) and these stimulatory effects could be notably suppressed by D1 agonist. These findings, taken together, suggest that the somatotrophs may serve as a major site of action for follistatin regulation by activin and GH as well as their functional interaction with dopaminergic input *via* D1 activation in the carp pituitary.

**Figure 8:**
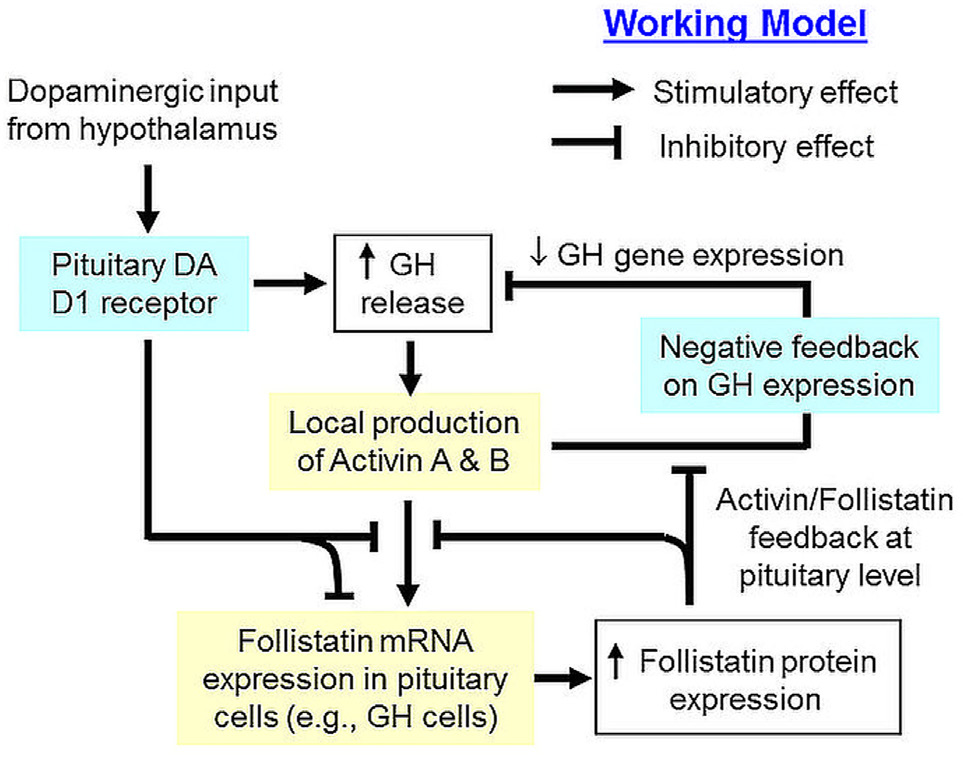
Working model of dopaminergic D1 interaction with activin/follistatin system for growth hormone (GH) regulation in the carp pituitary. In the carp pituitary, local release of GH can induce activin A and B production, which can exert a negative feedback to inhibit GH secretion and GH gene expression. Meanwhile, pituitary expression of follistatin, especially in carp somatotrophs, is also up-regulated by activin A and B, which can trigger a feedback inhibition to suppress the stimulatory effects of activin on follistatin and GH gene expression. Dopaminergic input from the hypothalamus, which is well-documented to stimulate GH release *via* activation of pituitary dopamine (DA) D1 receptor, can inhibit both basal as well as activin- and GH-induced follistatin expression at pituitary level. This DA D1 action presumably can relief activin feedback on GH regulation from the inhibitory effect of follistatin, which is a major component for signal termination of activin/follistatin system at tissue level.

In summary, grass carp follistatin has been cloned and found to be a single-copy gene in the carp genome. Its transcript expression has been demonstrated in the carp pituitary as well as in other tissues. At pituitary level, protein expression of follistatin, together with activin βA and βB, has been confirmed and follistatin transcript signals could be detected in carp somatotrophs, lactotrophs, and gonadotrophs. Functional expression also revealed that follistatin of carp origin could nullify activin action in transactivating target promoter with activin-responsive elements. Based on our studies with carp pituitary cells and enriched somatotrophs, follistatin has been confirmed to be a functional component for autocrine/paracrine regulation at the pituitary level and a working model for its role in activin/follistatin system related to GH regulation in carp pituitary has been proposed (Figure 8). In this model, activin A and B released locally induce follistatin mRNA expression in different pituitary cell types and subsequent production of follistatin, especially in carp somatotrophs, can exert a negative feedback to inhibit activin-induced follistatin expression. In carp pituitary cells, GH release is known to stimulate activin expression, which in turn can trigger an inhibitory feedback to suppress GH release and GH gene expression (42). However, the inhibitory effect of activin A and B on GH gene expression can also be negated by local production of follistatin, presumably caused by activin-induced follistatin expression. In carp species, dopaminergic input from the hypothalamus *via* DA D1 receptor activation is known to induce GH release at pituitary level (38). Interestingly, DA D1 activation in carp pituitary cells, especially in somatotrophs, can suppress both basal as well as activin- and GH-induced follistatin expression. These inhibitory actions mediated by DA D1 receptor presumably can help to relief the activin feedback on GH production from follistatin inhibition, which may fine tune the process of GH regulation by activin/follistatin system in the carp pituitary. Our study for the first time provide evidence that dopaminergic interaction with activin/follistatin system *via* D1 receptor expressed at pituitary level may form a new facet for GH regulation by autocrine/paracrine mechanisms in carp model.

## Ethics Statement

The study was conducted according to the recommended guidelines for the care and use of laboratory animals for research and teaching at the University of Hong Kong (Hong Kong).

## Author Contributions

AW and RF were responsible for project planning, data analysis, and manuscript writing. RF and BJ were involved in molecular cloning and functional studies. MH and KY were involved in LC/MS/MS and somatotroph preparation.

## Conflict of Interest Statement

The research was conducted in the absence of any commercial or financial relationships that could be construed as a potential conflict of interest.

## Abbreviations

GH, growth hormone; LH, luteinizing hormone; FSH, follicle-stimulating hormone; PRL, prolactin; hCG, human chorionic gonadotropin; DA, dopamine; APO, apomorphine; LCM, laser capture microdissection.
