# Supplementary material for: Grass Carp Follisatin: Molecular Cloning, Functional Characterization, Dopamine D1 Regulation at Pituitary Level, and Implication in Growth Hormone Regulation
Source: Front Endocrinol (Lausanne). 2017 Aug 24;8:211. doi: 10.3389/fendo.2017.00211 (PMC5574371; doi:10.3389/fendo.2017.00211)
Supplement: Supplementary file 1 [file Data_Sheet_1.PDF]

Supplemental Fig.1

1 ttcattcagcactgcctgagtaagaagattacttttgcgctgctcgtctcaatactctttctctctttaaac ATG CTA
M L

79 AAG ATG CTA AAG CGT CAG CAC CTC CAT CCG GGA ATG ATT TTA TTA CTC TTA TGG CTC TGT
R M L K R Q H L H P G M I L L L L W L C

Signal peptide

139 TAT TTG ATT GAA GAT CAA AAA GTG CAA GCT GGT AAC TGC TGG CTA CAG CAA GGC AAG AAC
Y L I E D Q K V Q A G N C W L Q Q G K N

N-terminal doamin

199 GGG AGA TGT CAG GTC CTC TAC ATG CCT GGG ATG AGT CGA GAG GAA TGC TGC CGG AGT GGG
G R C Q V L Y M P G M S R E E C C R S G

259 AGG CTC GGT ACA TCT TGG ACT GAG GAA GAT GTG CCA AAC AGC ACA TTA TTC AGG TGG ATG
R L G T S W T E E D V P N S T L F R W M

319 ATC TTC AAT GGC GGT GCT CCA AAC TGC ATA CCT TGT AAA GAG ACA TGC GAT AAT GTG GAC
I F N G G A P N C I P C K E T C D N V D

379 TGC GGC CCT GGG AAG AGA TGT AAA ATG AAC AGG AGG AGT AAG CCT CGC TGC GTC TGC GCC
C G P G K R C K M N R R S K P R C V C A

439 CCA GAC TGC TCC AAC ATC ACC TGG AAG GGG CCG GTG TGC GGC TCA GAC GGA AAA ACA TAC
P D C S N I T W K G P V C G S D G K T Y

FSD_1

499 CGA GAT GAA TGT GCC CTT TTG AAA TCC AAA TGC AAA GGG CAC CCG GAT CTG GAG GTG CAG
R D E C A L L K S K C K G H P D L E V Q

559 TAT CAA GGC AAA TGC AAA AAG ACG TGC CGT GAT GTC CTG TGT CCG GGA AGT TCG ACT TGT
Y Q G K C K K T C R D V L C P G S S T C

619 GTG GTG GAC CAG ACA AAC AAC GCG TAC TGT GTG ACA TGC AAC CGC ATA TGC CCA GAG GTT
V V D Q T N N A Y C V T C N R I C P E V

679 ACG TCT CCG GAT CAG TAC CTT TGT GGC AAC GAT GGG ATT GTT TAC GCC AGC GCG TGC CAT
T S P D Q Y L C G N D G I V Y A S A C H

FSD_2

739 TTA AGG AGA GCC ACG TGC TTG CTC GGC AGA TCC ATT GGT GTG GCA TAC GAA GGG AAA TGC
L R R A T C L L G R S I G V A Y E G K C

799 ATC AAG GCC AAG TCA TGC GAT GAT ATC CAG TGC AGC GTG GGG AAA AAG TGT CTA TGG GAT
I K A K S C D D I Q C S V G K K C L W D

859 GCC AAG ATG GGT CGC GGG CGG TGT GTG GTT TGT GTG GAG TCA TGC CCA GAA AGT CGC TCG
A K M G R G R C V V C V E S C P E S R S

919 GAG GAG GCC GTG TGC GCC AGC GAC AAC ACC ACA TAT CCC AGC GAG TGC GCC ATG AAG CAG
E E A V C A S D N T T Y P S E C A M K Q

FSD_3

979 GCC GCT TGC TCT TTG GGG GTT CTC CTG GAG GTT AAG CAT TTA GGA TCT TGC AAC TGT AAG
A A C S L G V L L E V K H L G S C N C K

1039 TAA tacatcttaaaagacaatccagtgctgcccccagcaacctcctccccaaaacctcccacccctccaaataacgcc
*

1117 tcttcattcccttcctgctgctaagagttaagctacagaaaggacttatgattggaagttctttgcatgtcctctttgg

1196 ctggcttcattaattttgcattttcttcag***aagaaa***aaaaatgttt***attaaa***aaagaacctacaaaatcaatggaaatc

1275 ***aatgaa***ttgaatc***aatgaa***aaaaaaaaaaaaaaaa

Supplemental Fig.1 Nucleotide and amino acid (a.a) sequences of grass carp follistatin The full-length cDNA of grass carp follistatin contains a 966 bp ORF encoding a 322 a.a. follistatin precursor. The ORF is presented in upper cases while the 5'UTR and 3'UTR are presented in lower cases. The signal peptide is marked with a dotted underline and the 4 structural domains identified in follistatin are underlined either in black line (for N-terminal domain) or with blue arrows (for FSD_{1-3} domains). The proteoglycan binding motif within the FSD_1 domain is boxed in red and the polyadenylation sites located in 3'UTR are underlined in italic for recognition.
