# Supplementary material for: Grass Carp Follisatin: Molecular Cloning, Functional Characterization, Dopamine D1 Regulation at Pituitary Level, and Implication in Growth Hormone Regulation
Source: Front Endocrinol (Lausanne). 2017 Aug 24;8:211. doi: 10.3389/fendo.2017.00211 (PMC5574371; doi:10.3389/fendo.2017.00211)
Supplement: Supplementary file 2 [file Data_Sheet_2.PDF]

Supplemental Fig.2

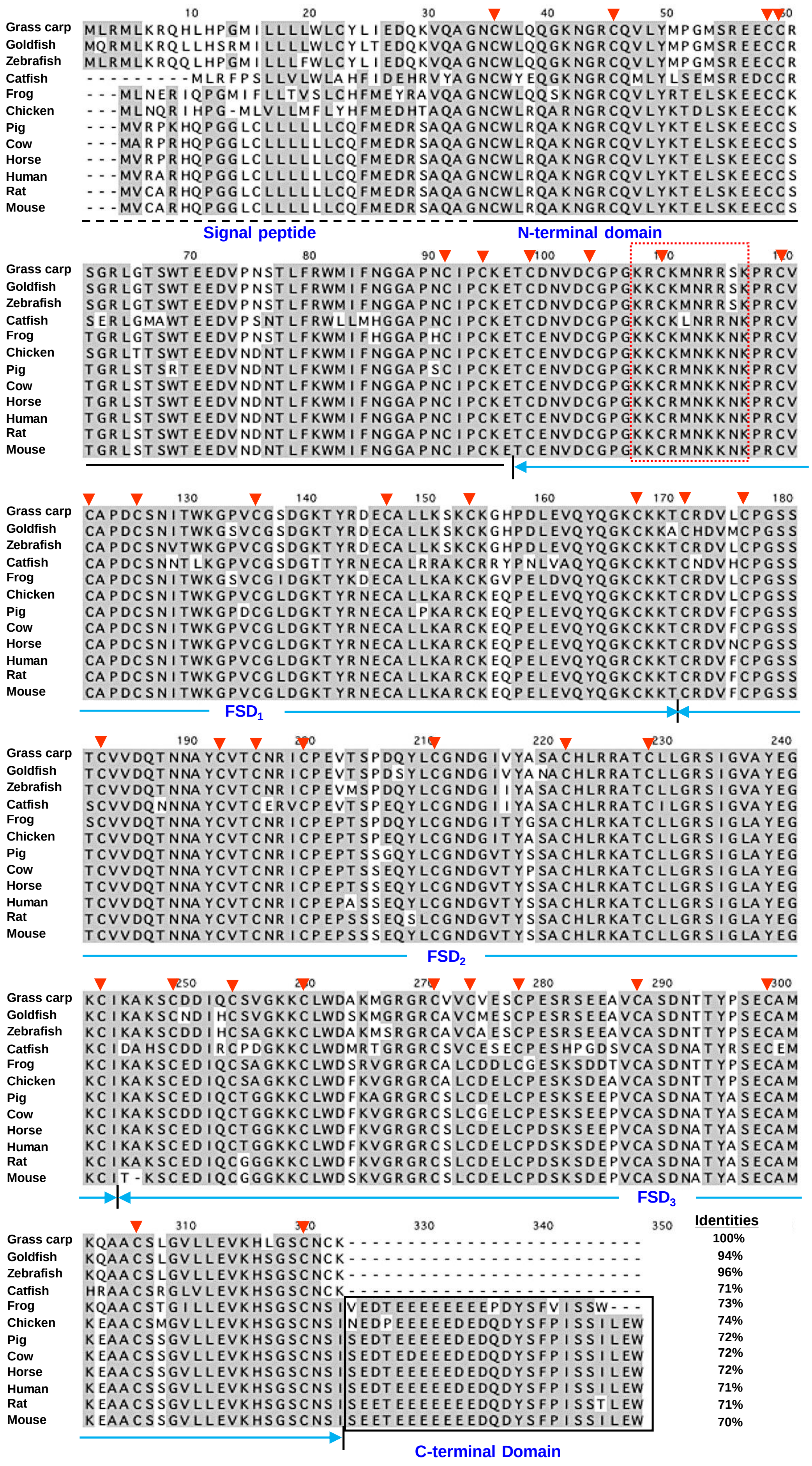

Supplemental Fig.2   Alignment of the deduced protein sequence of grass carp follistatin with corresponding sequences reported in other vertebrates. The sequence alignment was conducted using Clustal W algorithm. The conserved residues within the protein sequences are boxed in gray. The signal peptide is marked by dotted underline while the 4 structural domains identified in carp follistatin are underlined either in black line (for N-terminal domain) or with blue arrows (for FSD_{1-3} domains). The proteoglycan binding motif within the FSD_1 domain is boxed in red while the 36 conserved cysteine residues for intramolecular disulfide bonding are marked by red triangles. Of note, the C-terminal domain coding for C-terminal tail in tetrapod follistatin is missing in the corresponding region in fish model. The percentage of sequence homology of carp follistatin compared with the corresponding sequences of other species is listed at the end of individual sequences.
