# Supplementary material for: Grass Carp Follisatin: Molecular Cloning, Functional Characterization, Dopamine D1 Regulation at Pituitary Level, and Implication in Growth Hormone Regulation
Source: Front Endocrinol (Lausanne). 2017 Aug 24;8:211. doi: 10.3389/fendo.2017.00211 (PMC5574371; doi:10.3389/fendo.2017.00211)
Supplement: Supplementary file 3 [file Data_Sheet_3.PDF]

Supplemental Fig.3

Peptide fragments identified for grass carp follistatin

A Follistatin (Protein coverage by peptides identified in B: 57.8%, grass carp pituitary)

Peptide 1

MLRMLKRQHLHPGMILLLLWLCYLIEDQKVQAGNCWLQQGKNGRCQVLYMPGMSREECCRSGRLGTSWTEEDVPNSTLFRWMIF

Peptide 2

NGGAPNCIPCKETCDNVDCGPGKRCKMNRRSKPRCVCAPDCSNITWKGPVCGSDGKTYRDECALLKSKCKGHPDLEVQYQGKCK

KTCRDVLCPGSSTCVVDQTNNAYCVTCNRICPEVTSPDQYLCGNDGIVYASACHLRRATCLLGRSIGVAYEGKCIKAKSCDDIQ

Peptide 3

CSVGKKCLWDAKMGRGRCVVCVESCPESRSEEAVCASDNTTYPSECAMKQAACSLGVLLEVKHLGSCNCK

B Peptides identified with >95% confidence

Confid.	Peptide Sequence	ΔMass	Obs MW	z	Obs m/z
99	AKSCDDIQCSVGK	0.019	2357.22	3	786.75
99	ATCLLGR	0.027	1082.62	2	542.32
99	ATCLLGRSIGVAYEGK	0.103	2291.36	4	573.83
99	ATCLLGRSIGVAYEGKCIK	-0.179	2985.46	4	747.37
99	CKGHPDLEVQYQGK	-0.066	2559.30	4	640.83
99	CLWDAK	0.033	1388.77	3	695.38
99	DECALLKSKCK	0.052	2545.47	4	637.37
99	ETCDNVDCGPGK	0.063	1936.92	2	969.47
99	ETCDNVDCGPGKR	0.038	2093.00	4	524.26
99	GHPDLEVQYQGK	0.131	1978.20	4	495.56
99	GPVCGSDGK (Peptide 2)	0.081	1472.84	2	737.40
99	GPVCGSDGKTYRDECALLK	0.194	3015.74	4	754.94
99	LEVK	-0.002	1095.71	2	548.86
99	LGTSWTEEDVPNSTLFR	0.085	2255.22	3	752.75
99	LRMLK	-0.072	1267.75	2	423.61
99	MLRMLK	-0.085	1414.78	2	708.40
99	NGRCQVLYMPGMSR	0.055	1961.00	2	981.51
99	QAACSLGVLLEVK (Peptide 3)	-0.017	1984.11	3	662.38
99	RATCLLGRSIGVAYEGK	-0.110	2447.25	3	816.76
99	SIGVAYEGK	-0.080	1530.81	2	766.42
99	SIGVAYEGKCIK	-0.117	2225.15	3	557.33
99	TYRDECALLK	-0.011	1864.99	3	622.67
99	TYRDECALLKSK	-0.117	2384.22	3	795.75
99	VQAGNCWLQQGK	-0.009	1986.02	3	663.01
99	VQAGNCWLQQGKNGR (Peptide 1)	-0.007	2312.20	3	771.77
99	VQAGNCWLQQGKNGRCQVLYMPG MSR	0.068	3623.84	4	906.97
99	WMIFNGGAPNCIPCK	0.104	2350.24	3	784.42
97.9	DECALLK	-0.012	1444.78	2	723.40
96.4	QHLHPGMILLLLWLCYLIEDQK	-0.047	3008.55	4	753.14
95.5	CLWDAKMGRGR	-0.031	1961.99	2	982.00

C Representative mass spectra

Peptide 1

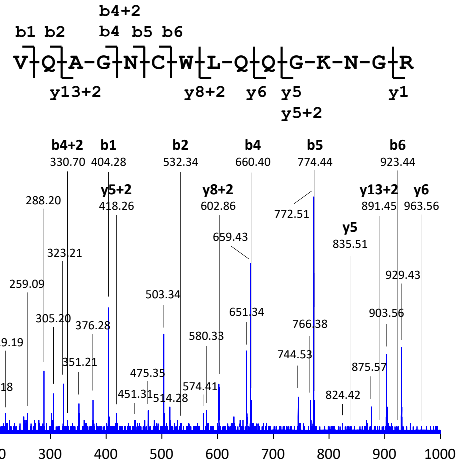

Peptide 2

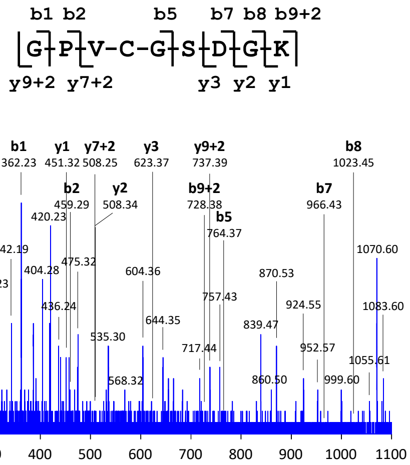

Peptide 3

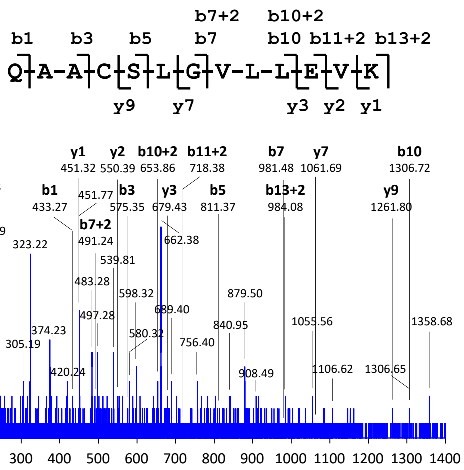

Supplemental Fig.3 Protein expression of follistatin in carp pituitary detected by proteomic approach. Protein lysate was prepared from the carp pituitary and subjected to trypsin digestion followed by LC/MS/MS. Digested products were resolved by C_{18} chromatography followed by MS/MS detection using a SCIEX TripleTOF 5600 system. Peptide products originated from carp follistatin were identified by ProteinPilot 2.0 and the regions in target protein covered by these peptides with different confidence levels were color-coded as shown in panel (A), with green color for the regions with confidence ≥ 95%, red color for confidence between 95% and 50%, and yellow color for confidence between 50% and 20%. Sequences of follistatin peptides identified with confidence level ≥ 95% as well as the corresponding QC data, including confidence score (Confid.) and mass derivation (Δ Mass), and MS data, including the precise molecular weight (Prec MW), theoretical charge (Z) and precise mass-to-charge ratio (Prec m/z), are presented in panel (B) and their respective locations within follistatin sequence were marked by black underscores in panel (A). For follistatin protein expression at pituitary level, representative MS spectra for peptide fragments identified, designated as peptide 1, 2 and 3, are presented in panel (C) and their respective position in follistatin sequence is marked by yellow shading. In individual spectra, the ion peaks corresponding to the series of b- and y-fragments generated by collision-induced fragmentation were also annotated for the respective peptides.
