# Supplementary material for: Grass Carp Follisatin: Molecular Cloning, Functional Characterization, Dopamine D1 Regulation at Pituitary Level, and Implication in Growth Hormone Regulation
Source: Front Endocrinol (Lausanne). 2017 Aug 24;8:211. doi: 10.3389/fendo.2017.00211 (PMC5574371; doi:10.3389/fendo.2017.00211)
Supplement: Supplementary file 4 [file Data_Sheet_4.PDF]

Supplemental Fig.4

Peptide fragments identified for grass carp activin βA

A Activin βA (Protein coverage by peptides identified in B: 56.9%, grass carp pituitary)

MSSLTLVTGVLLLSGCLSGGCSPTPAESGSQGAGHPDDPAVTPCPSCALAQRQKDSEEQTDMVEAVKRHILNMLHLNTRPNVTH

Peptide 1

PVPRAALLNAIRKLHVGRVGEDGTVEIEEDGGGLGEREQPEEQPFEIITFAEPGDAPDVMKFDISKEGNTLSVVEQANVWLFLK

Peptide 2

VAKGNRGKGKVSIQLLQHGKADPVSTDGSQELVVSEKTVDTRRSGWHTLPVPRMVQTLLDGDSSFLSLRVSCPLCAEAGAVPIL

Peptide 3

VPAEGNKGKEREQSHRPFLMVVLKPAEEHQHRRSKRGLECDGKIRVCCKRQFYVNFKDIGWSDWIIAPSGYHANYCEGDCPSHV

ASITGSALSFHSTVINHYRMRGYSPFTNIKSCCVPTRLRAMSMLYYNEEQKIIKKDIQNMIVEECGCS

B Peptides identified with >95% confidence

Confid.	Peptide Sequence	ΔMass	Obs MW	z	Obs m/z
99	AALLNAIR	0.004	1145.71	2	573.86
99	AALLNAIRK (Peptide 1)	0.088	1349.92	3	450.98
99	ADPVSTDGSQELVVSEK	0.029	2368.29	3	790.44
99	AMSMLYYNEEQK	0.143	2115.19	3	706.07
99	AMSMLYYNEEQKIIK	-0.025	2772.51	4	694.13
99	DSEEQTDMVEAVK	0.040	2088.09	3	697.04
99	EGNTLSVVEQANVWLFLK	0.004	2332.28	4	584.08
99	EQPEEQPFEIITFAEPGDAPDVMK	0.145	3325.80	4	832.46
99	FDISK	0.002	1216.73	2	609.37
99	GKER	-0.020	1096.66	2	549.34
99	GKVSIQLLQHGK	0.000	2220.37	4	556.10
99	GLECDGK	0.129	1374.84	3	688.38
99	GLECDGKIRVCCK	0.119	2473.38	3	825.47
99	GNRGK	0.035	1138.74	2	570.38
99	GNRGKGK (Peptide 2)	-0.032	1628.98	3	544.00
99	GYSPFTNIKSCCVPTR	0.013	2472.24	3	825.09
99	IIKK	-0.015	1412.97	3	472.01
99	IRVCCK	0.096	1420.86	3	711.38
99	IRVCCKR	-0.037	1576.83	2	789.42
99	LHVGRVGEDGTVEIEEDGGGLGER	-0.029	2783.37	4	696.85
99	LRAMSMLYYNEEQK	0.120	2383.37	3	795.46
99	LRAMSMLYYNEEQKIIK	-0.006	3057.71	4	765.43
99	MRGYSPFTNIK	0.095	1937.16	4	485.30
99	MRGYSPFTNIKSCCVPTR	0.009	2759.38	3	920.80
99	QFYVNFK	0.081	1553.95	3	518.99
99	QKDSEEQTDMVEAVK	-0.089	2649.30	4	663.33
99	RGLECDGK	0.022	1530.83	3	511.27
99	RQFYVNFK (Peptide 3)	0.002	1708.99	2	570.67
99	RSGWHTLPVPR	-0.036	1608.88	2	805.42
99	SGWHTLPVPR	0.048	1452.86	2	727.38
99	TVDTR	-0.002	894.51	2	448.26
99	TVDTRR	0.020	1050.63	2	526.32
99	TVDTRRSGWHTLPVPR	-0.084	2181.12	3	728.05
99	VAKGNR	-0.068	1251.72	2	626.87
99	VAKGNRGK	-0.083	1741.03	3	581.35
99	VCCKRQFYVNFK	-0.116	2538.23	3	847.08
99	VGEDGTVEIEEDGGGLGER	0.171	2221.24	4	556.32
99	VSIQLLQHGK	-0.128	1729.94	3	577.65
99	VSIQLLQHGKADPVSTDGSQELVVSEK	-0.156	3775.95	5	756.20
98.5	AALLNAIRKLHVGR	-0.171	2139.19	4	535.80
96.8	AESGSQGAGHP	0.027	1800.02	3	601.02
96.1	EQSHRPFLMVVLKPAEEHQHR	-0.057	2853.46	3	952.18
95.3	VCCK	0.063	1151.64	2	576.83

C Representative mass spectra

Peptide 1

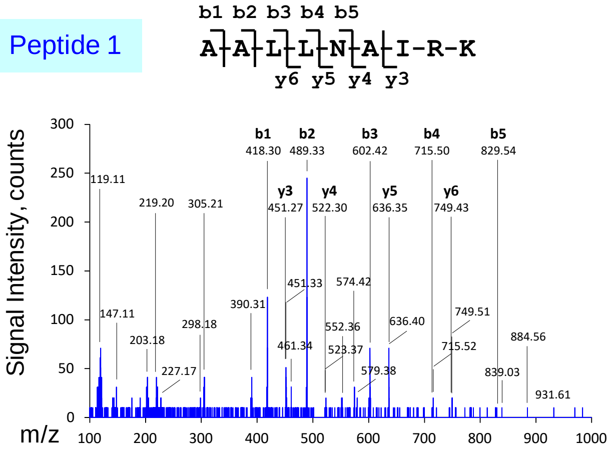

Peptide 2

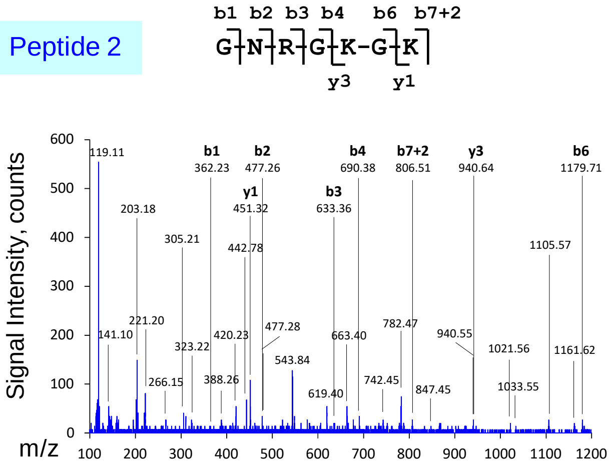

Peptide 3

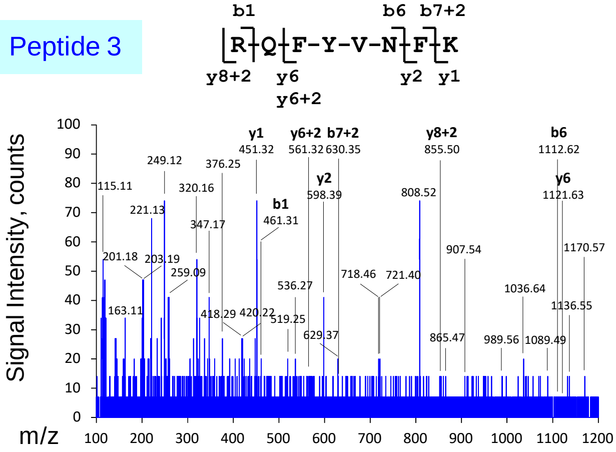

Supplemental Fig.4 Protein expression of activin βA in carp pituitary detected by proteomic approach. Protein lysate was prepared from the carp pituitary and subjected to trypsin digestion followed by LC/MS/MS. Digested products were resolved by C_{18} chromatography followed by MS/MS detection using a SCIEX TripleTOF 5600 system. Peptide products originated from carp activin βA were identified by ProteinPilot 2.0 and the regions in target protein covered by these peptides with different confidence levels were color-coded as shown in panel (A), with green color for the regions with confidence ≥ 95%, red color for confidence between 95% and 50%, and yellow color for confidence between 50% and 20%. Sequences of activin βA peptides identified with confidence ≥ 95% as well as the corresponding QC data, including confidence score (Confid.) and mass derivation (Δ Mass), and MS data, including the precise molecular weight (Prec MW), theoretical charge (Z) and precise mass-to-charge ratio (Prec m/z), are presented in panel (B) and their respective locations within activin βA sequence were marked by black underscores in panel (A). For protein expression of activin βA at the pituitary level, representative MS spectra for peptide fragments identified, designated as peptide 1, 2 and 3, are presented in panel (C) and their corresponding position in activin βA sequence is marked by yellow shading. In individual spectra, the ion peaks corresponding to the series of b- and y-fragments generated by collision-induced fragmentation were also annotated for the respective peptides.
