# Supplementary material for: Grass Carp Follisatin: Molecular Cloning, Functional Characterization, Dopamine D1 Regulation at Pituitary Level, and Implication in Growth Hormone Regulation
Source: Front Endocrinol (Lausanne). 2017 Aug 24;8:211. doi: 10.3389/fendo.2017.00211 (PMC5574371; doi:10.3389/fendo.2017.00211)
Supplement: Supplementary file 5 [file Data_Sheet_5.PDF]

Supplemental Fig.5

Peptide fragments identified for grass carp activin βB

A Activin βB (Protein coverage by peptides identified in B: 59.4%, grass carp pituitary)

MDTLFKKMSIYILSVTCLMACILSVQCSLGAETVSQESQCVSCGLGHSDDSGRMDTDFLEAVKRHILNRLQLRERPNITHPIPK

Peptide 1
AAMVTALRKLHAGKVREDGRVEIPNLDGHAAYNEVQEETSEIISFAESDDVTPSKSSLYFLISNEGNQNLYVLQANLWLYFKLL

Peptide 2
PGAQEKGLRRKVTVRVHYYEPGGQNMHWPVMEKRVELKRSGWHTFPVSEAVREMLAKGGRRQDLDIHCEGCEAANVLPILVDPS

Peptide 3
DPSHRPFLVVRAQQAEGKHRIRKRGLECDGNNGGLCCRQQFYIDFRLIGWNDWIIAPAGYYGNYCEGSCPAYMAGVPGSASSFH

TAVVNQYRMRGMSPGSVNSCCIPTKLSTMSMLYFDDEYNIVKRDVPNMIVEECGCA

B Peptides identified with >95% confidence

Confid.	Peptide Sequence	ΔMass	Obs MW	z	Obs m/z
99	AAMVTALR	0.009	1135.68	2	568.83
99	AAMVTALRK (Peptide 1)	-0.052	1567.92	3	523.65
99	AQQAEGK	-0.028	1339.73	2	670.87
99	AQQAEGKHR (Peptide 3)	0.105	1633.02	3	545.35
99	DTLFK	-0.013	1230.73	3	616.35
99	DTLFKK	-0.112	1662.93	3	555.32
99	EMLAK	0.008	1198.73	3	400.58
99	EMLAKGGRR	-0.056	1624.91	2	813.44
99	ERPNITHPIPK	-0.082	1909.05	3	637.35
99	ERPNITHPIPKAAMVTALR	-0.132	2722.46	4	681.62
99	GLRRK	-0.093	1236.73	2	619.37
99	GMSPGSVNSCCIPTK	0.012	2180.05	3	727.69
99	KLHAGK	-0.087	1564.93	3	522.66
99	LHAGK	-0.041	1132.68	2	567.35
99	LLPGAQEK (Peptide 2)	-0.063	1462.83	3	488.62
99	LLPGAQEKGLRR	-0.091	1945.11	4	487.29
99	LQLR	0.006	833.53	2	417.77
99	LQLRERPNITHPIPK	-0.098	2419.37	3	605.87
99	LSTMSMLYFDDEYNIVK	-0.050	2677.30	4	893.47
99	LSTMSMLYFDDEYNIVKR	0.074	2832.54	4	709.14
99	MDTDFLEAVK	-0.049	1791.90	2	598.34
99	MDTDFLEAVKR	0.089	1932.15	4	484.04
99	MDTLFK	0.000	1361.78	3	454.94
99	MRGMSPGSVNSCCIPTK	0.073	2467.25	3	823.42
99	QQFYIDFR	0.016	1420.75	2	711.38
99	RHILNR	-0.060	1111.63	2	556.82
99	RHILNRLQLR	-0.110	1621.91	3	541.64
99	RSGWHTFPVSEAVR	0.112	1932.14	3	484.04
99	RVELK	0.042	1251.85	3	418.29
99	SGWHTFPVSEAVREMLAK	0.054	2652.48	3	885.17
99	VELK	0.000	1095.71	2	548.86
99	VHYYEPGGQNMHWPVMEK	0.064	2809.46	4	703.37
99	VREDGR	0.056	1034.63	2	518.32
99	VSEAVR	-0.005	1239.76	2	620.89
98.3	LQLRERPNITH	-0.045	1679.93	2	840.97
96.7	QDLDIHCEGCEAANVLPILVDPSDP SHRPFLVVR	0.133	3828.94	4	958.24
96.1	REMLAK	-0.011	1354.81	2	678.41
95.5	LSVQCSLGAETV	0.056	1555.84	2	778.92

C Representative mass spectra

Peptide 1

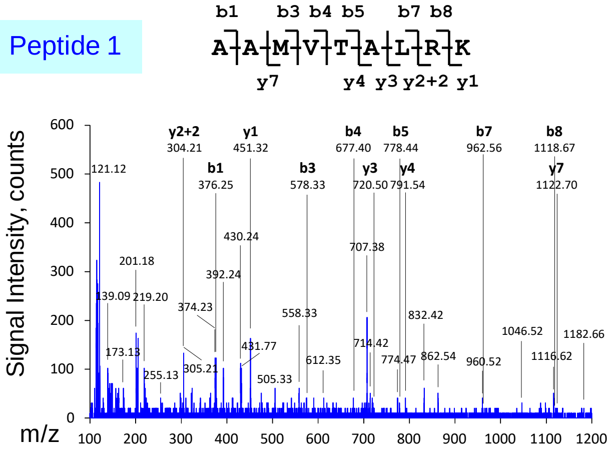

Peptide 2

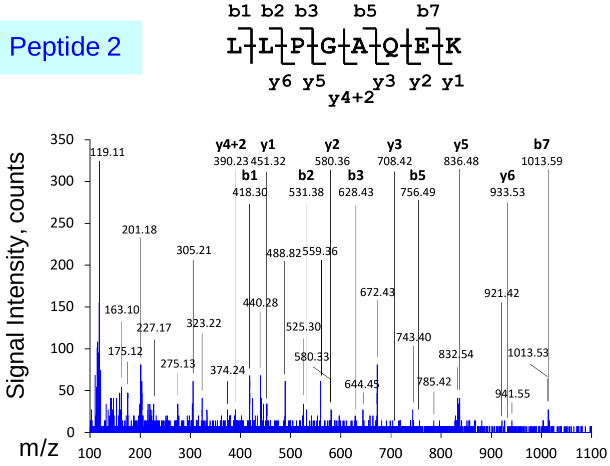

Peptide 3

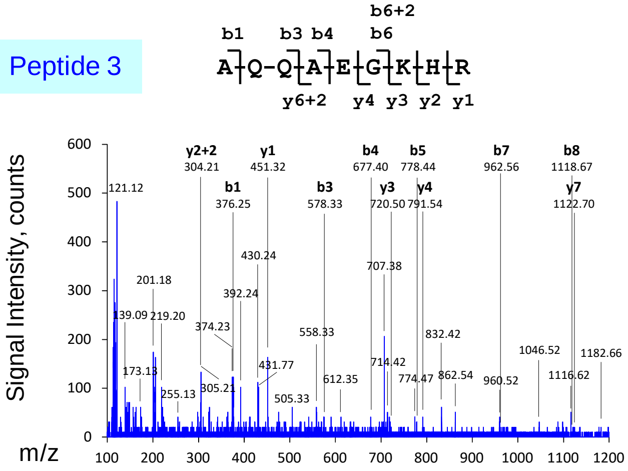

<u>Supplemental Fig.5</u> Protein expression of activin βB in carp pituitary detected by proteomic approach. Protein lysate was prepared from the carp pituitary and subjected to trypsin digestion followed by LC/MS/MS. Digested products were resolved by C_{18} chromatography followed by MS/MS detection using a SCIEX TripleTOF 5600 system. Peptide products originated from carp activin βB were identified by ProteinPilot 2.0 and the regions in target protein covered by these peptides with different confidence levels were color-coded as shown in panel (A), with green color for the region with confidence ≥ 95%, red color for confidence between 95% and 50%, and yellow color for confidence between 50% and 20%. Sequences of activin βB peptides identified with confidence ≥ 95% as well as the corresponding QC data, including confidence score (Confid.) and mass derivation (Δ Mass), and MS data, including the precise molecular weight (Prec MW), theoretical charge (Z) and precise mass-to-charge ratio (Prec m/z), are presented in panel (B) and their respective locations within activin βB sequence were marked by black underscores in panel (A). For protein expression of activin βB at the pituitary level, representative MS spectra for peptide fragments identified, designated as peptide 1, 2 and 3, are presented in panel (C) and their corresponding position in activin βB sequence is marked by yellow shading. In individual spectra, the ion peaks corresponding to the series of b- and y-fragments generated by collision-induced fragmentation were also annotated for the respective peptides.
